# Synthesis, Electrochemistry and Density Functional Theory of Osmium(II) Containing Different 2,2′:6′,2″-Terpyridines

**DOI:** 10.3390/molecules29215078

**Published:** 2024-10-27

**Authors:** Nandisiwe G. S. Mateyise, Marrigje M. Conradie, Jeanet Conradie

**Affiliations:** 1 Department of Chemistry, University of the Free State, P.O. Box 339, Bloemfontein 9300, South Africa; nmateyise@gmail.com; 2 Department of Chemistry, UiT—The Arctic University of Norway, N-9037 Tromsø, Norway

**Keywords:** osmium(II), redox potential, electrochemistry, DFT, terpyridine

## Abstract

In coordination chemistry, 2,2′:6′,2″-terpyridine is a versatile and extensively studied tridentate ligand. Terpyridine forms stable complexes with a variety of metal ions through coordination sites provided by the three nitrogen atoms in its pyridine rings. This paper presents an electrochemical study on various bis(terpyridine)osmium(II) complexes, addressing the absence of a systematic investigation into their redox behavior. Additionally, a computational chemistry analysis was conducted on these complexes, as well as on eight previously studied osmium(II)-bipyridine and -phenanthroline complexes, to expand both the experimental and theoretical understanding. The experimental redox potentials, Hammett constants, and DFT-calculated energies show linear correlations due to the electron-donating or electron-withdrawing nature of the substituents, as described by the Hammett constants. These substituent effects cause shifts to lower or higher redox potentials, respectively.

## 1. Introduction

In coordination chemistry, 2,2′:6′,2″-terpyridine, first reported in 1932 [1], is a versatile and widely studied tridentate ligand. Its structure consists of three pyridine rings connected by carbon–carbon bonds, forming a conjugated system. The three nitrogen atoms in the pyridine rings provide coordination sites, allowing terpyridine to form stable complexes with a variety of metal ions [2]. Terpyridine is favored as a ligand for synthesizing various metal compounds due to its strong binding affinity to metal centers. The nitrogen atoms typically occupy adjacent coordination sites on the metal, resulting in a highly stable chelate effect. Due to this stability and terpyridine’s electronic properties, the resulting metal complexes exhibit unique qualities that make them valuable in a wide range of applications, including catalysis [2,3,4], supramolecular chemistry [5,6], materials science [7], optoelectronics [8], photovoltaics [9], medicine [2,10,11], antimicrobial agents [12], chemosensors [2], photocatalysis [2,3,13], photosensitizers [2,14,15], light-emitting diodes (LEDs) [8], and as building blocks for molecular magnetic materials [16,17].

Osmium is the densest and rarest stable element in the earth’s crust, with an estimated concentration of about 0.00005 ppm. This scarcity, along with the high cost of commonly used precursors such as OsCl₃, may explain why the coordination and organometallic chemistry of osmium is less developed and less frequently reported in the literature compared to other group 8 transition metals like iron and ruthenium [18]. However, due to their wide range of applications and unique properties, bis(terpyridine)osmium complexes have attracted attention from chemists over the past decades. In these complexes, two terpyridine ligands coordinate to an osmium center, forming a robust metal–ligand framework. Terpyridine ligands are commonly coordinated to osmium(II) and osmium(III) precursors during synthesis, yielding complexes with distinct structural and electronic characteristics. The study of terpyridine containing osmium complexes as catalysts for various chemical transformations, such as oxidation reactions, has been a focus of interest. These complexes serve as effective catalysts in organic synthesis and energy conversion applications due to their tunable electronic properties and ability to undergo reversible redox reactions [19,20]. The intriguing photophysical properties of bis(terpyridine)osmium complexes make them attractive candidates for optoelectronic devices [20]. By modifying the coordination environment around the osmium center or the terpyridine ligands, their absorption and emission characteristics can be tailored. Osmium(II) polypyridyl complexes have been investigated for applications in sensors [21], photovoltaic devices [10,22], and light-emitting diodes (LEDs) [23,24]. Dye-sensitized solar cells (DSSCs) are a notable example of electrochemical energy conversion devices in which these complexes serve as efficient photosensitizers [22,25,26]. The ability of a compound to function as a redox mediator in DSSCs is determined by its redox potentials [27]. As a result, conducting an electrochemical study on a series of polypyridine Os complexes could be a preliminary step toward assessing their potential as future redox mediators.

**Scheme 1:**
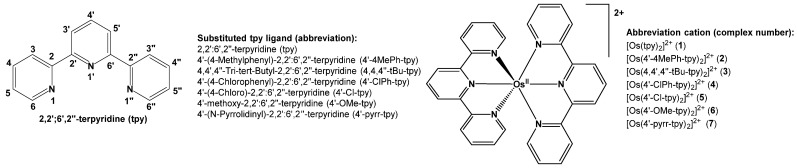
Structure of ligands and complexes synthesized for this work. The abbreviation used for the ligands and complex numbers are given.

**Scheme 2:**
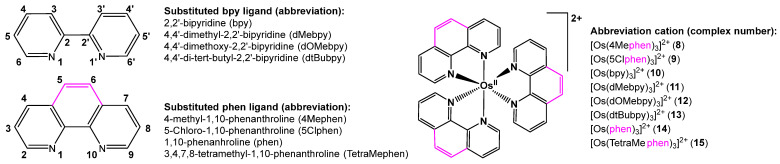
Structure of ligands and six-coordinated octahedral tris(polypyridine)osmium(II) complexes included in the theoretical study in this work. The abbreviations used for the ligands and complex numbers are given. Although four-coordinated tetrahedral bis(polypyridine)iron(II) exists [33], no four-coordinated bis(polypyridine)osmium(II) could be found in the literature.

This study presents an electrochemical investigation using cyclic voltammetry (CV) on various six-coordinated, distorted octahedral bis(terpyridine)osmium(II) complexes (**1**)–(**7**), as outlined in Scheme 1. The study was necessary due to the lack of a systematic investigation into the redox behavior of bis(terpyridine)osmium(II) complexes. Additionally, a computational chemistry analysis was performed on complexes (**1**)–(**7**), as well as on a series of six-coordinated octahedral tris(polypyridine)osmium(II) complexes (**8**)–(**15**), whose redox properties have been previously documented under the same experimental conditions [28], as shown in Scheme 2. This was carried out to further enrich both the experimental and theoretical aspects of the study. While mixed-ligand bis(terpyridine)osmium(II) complexes containing different substituted terpyridine ligands are reported [29,30,31,32], this study focuses on bis(terpyridine)osmium(II) and tris(polypyridine)osmium(II) complexes that contain only two identical tridentate or three identical bidentate ligands, as illustrated in Scheme 1 and Scheme 2, respectively.

## 2. Results and Discussion

A selection of seven bis(terpyridine)osmium(II) complexes (**1**)–(**7**) were experimentally synthesized and characterized. An experimental electrochemical study using cyclic voltammetry was performed on the complexes. A theoretical DFT study was conducted on (**1**)–(**15**) in order to establish relationships between experimentally measured redox potential and DFT-calculated energies and charges for the larger series of polypyridine-osmium(II) complexes (**1**)–(**15**).

### 2.1. Synthesis

**Scheme 3:**
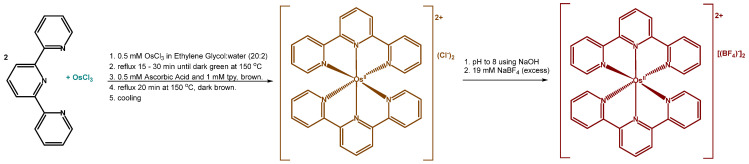
Synthesis of bis(tpy)-osmium(II), [Os(2,2′:6′,2″-terpyridine)_2_](BF_4_)_2_.

The synthesis scheme of bis(terpyridine)osmium(II) complexes (**1**)–(**7**) in Scheme 1 is portrayed in Scheme 3. Os(III) in OsCl_3_ was reduced to Os(II) by the high boiling point solvent, ethylene glycol [34], as well as ascorbic acid. The tpy ligands were then reacted with Os(II) to produce [Os(tpy)_2_](Cl)_2_. After cooling, an excess of NaBF_4_ was added to the mixture to obtain the dark brown product [Os(tpy)_2_](BF_4_)_2_.

### 2.2. CV Results

**Scheme 4:**
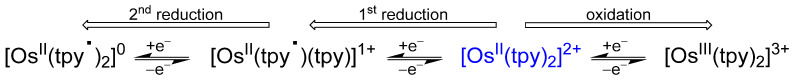
Oxidation and reduction of [Os(tpy)_2_]^2+^.

The oxidation and reduction of osmium(II) coordinated to different 2,2′:6′,2″-terpyridines have been reported [19,20,25,32,35,36,37,38,39]. The oxidation of the molecules is reported to be metal-based [19], while the reduction of the complexes is reported to occur on the coordinated polypyridine ligands [19,32], as illustrated in Scheme 4. Earlier reports wrongly assigned the reduction to Os(II/I) [20,35,37] and Os(I/0) [20,37]. Only a few cyclic voltammograms (CVs), at only one scan rate (ν), were provided in the literature [32,38,39,40,41]. Some benefits of obtaining redox data and corresponding cyclic voltammograms at varying scan rates are (i) to confirm that the electrochemical behavior observed at 0.100 V s⁻¹ remains consistent at higher scan rates, (ii) to assess whether the reduction process is diffusion-controlled by applying the Randles–Ševčík equation, and (iii) to calculate the diffusion coefficients of the analyte, which is crucial for understanding the reactivity of the species being studied, also through the application of the Randles–Ševčík equation. Therefore, this section presents an electrochemical study using CV experiments on a series of bis(terpyridine)Os(II) complexes (**1**)–(**7**), at a series of scan rates ν = 0.02–5.00 Vs^−1^.

The electrochemical data obtained at a scan rate of 0.100 Vs^−1^ for (**1**)–(**7**) are summarized in Table 1 (oxidation), Table 2 (1st reduction), and Table 3 (2nd reduction), together with previously reported data from the literature. The results obtained in this work for complexes (**1**) and (**2**) agree well with available experimental results.

**Figure 1:**
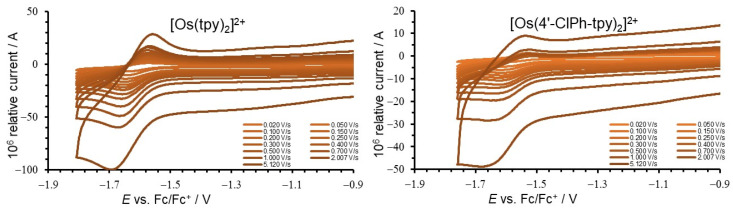
CVs of the reduction of (**1**) and (**4**) in CH_3_CN, at the indicated scan rates.

**Figure 2:**
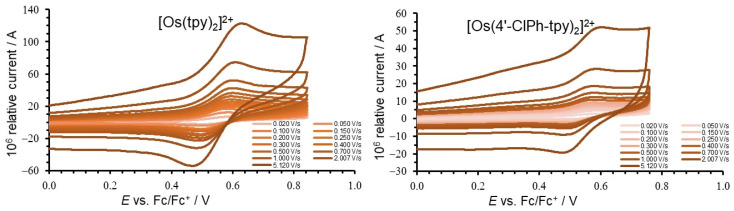
CVs of the oxidation of (**1**) and (**4**) in CH_3_CN, at the indicated scan rates.

**Figure 3:**
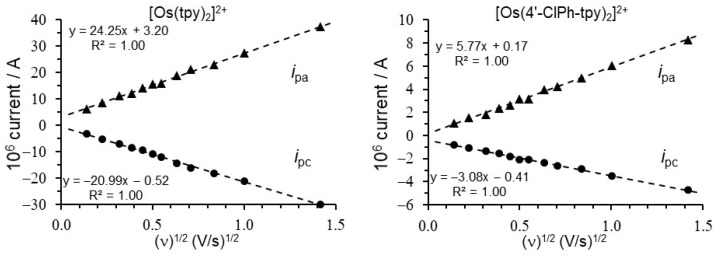
The linear relationship between peak currents (*i_p_*) and (scan rate)^1/2^ of the oxidation of (**1**) and (**4**).

The cyclic voltammograms indicating the reduction of complexes (**1**) and (**4**) are shown in Figure 1, where each displays a reduction peak at a potential of less than −1.5 V versus Fc/Fc^+^. Figure 2 shows the cyclic voltammograms of the oxidation of complexes (**1**) and (**4**), which are distinguished by their peak current potential separation (∆E_p_) of 0.059–0.089 V, see data in Table 1. Both complexes exhibit an oxidation potential of less than 0.6 V versus Fc/Fc^+^. The graph of the peak currents of the oxidation couple versus the square root of the scan rate is shown in Figure 3.

For a redox couple to exhibit electrochemical reversibility according to Nernstian behavior, a peak current ratio of one, as well as a Δ*E*_p_ value of 0.059 V (for a single electron transfer process), is necessary [43,44]. Ferrocene under the same experimental conditions used here has Δ*E*_p_ up to 0.080 V. Factors such as slow electron-transfer kinetics, imperfections in the experimental setup, or the effects of ohmic drop may lead to an increased Δ*E*_p_ and smaller current ratios [45,46,47]. The peak currents of the oxidation peak demonstrate a direct proportionality to the square root of the scan rate, see Figure 3, suggesting a diffusion controlled Os(II/III) oxidation [43]. Complexes containing electron withdrawing substituent groups on tpy (pyrrole, 0.051 V) exhibit a significantly lower oxidation potential (*E*_1/2_) when compared to the complexes containing tpy ligands with electron donating substituent groups (^t^Bu (0.394 V), 4′-4MePh (0.495 V)), see Table 1.

While the oxidation of (**1**)–(**7**) varies over a range of potentials of ca 0.5 V, the reduction varies over a decreased range of potentials, implying that the electronic influence of the substituents on tpy is more pronounced on the metal (osmium, oxidation center) than on the tpy ligand (reduction center). The peak current potential difference (∆*E*_p_) of the 1st reduction is less than 0.09 V, while a slightly larger ∆E_p_ is generally obtained for the 2nd reduction peak.

### 2.3. Theoretical Calculations

This division presents DFT results for [Os(tpy)_2_]^m^ for m = 0–3, including information on the ground-state geometries and electronic structure. DFT computations in the solvent phase (acetonitrile, the experimental solvent for redox potentials) were used to determine the complexes’ lowest energy geometries. Using solid-state crystallography, three of [Os(tpy)_2_]^2+^ molecules, besides eight other [Os(tpy)_2_]^2+^ complexes with modified terpyridine ligands (other than that of complexes **2**–**7**), were found on the Cambridge Structural Database [48]. No crystal structures could be found for [Os(tpy)_2_]^m+^ with m = 0, 1 or 3.

#### 2.3.1. Geometry of Bis(terpyridine)osmium(II) (**1**)–(**7**)

Table 4 presents our B3LYP/6-311(d,p)/def2tzvpp-computed geometrical results for (**1**)–(**7**) along with geometrical results obtained from published experimental solid-state X-ray structures for (**1**). The calculated complexes (**1**) and (**5**) converged to *D*_2d_ symmetry. The four terminal Os-N bonds were of equal length under the *D*_2d_ symmetry for (**1**) and (**5**). Similarly, the two Os-N central bonds were of equal length under the *D*_2d_ symmetry for (**1**) and (**5**). The four equal terminal bonds (Os-N_terminal_, represented by BL1, BL3, BL4, and BL6) were longer than the two equal central bonds (Os-N_central_, represented by BL2 and BL5, defined in Table 4), because of the strain caused by the tridentate terpyridine ligand. The substituents on the ligands reduced the symmetry of the molecule to *C*_2_ for (**4**), (**6**), and (**7**), to *C*_2v_ for (**3**), and to no symmetry for (**2**). The Os-N bonds in Table 4 show that each molecule’s Os(tpy)_2_ core was still close to *D*_2d_ symmetry, which meant that each molecule’s four terminal and two central bond lengths remained constant within the bounds of experimental error. The angles in these pseudo-octahedral complexes (about 78° and 92°) differ from 90° as in a true octahedron due to the strain in the tridentate tpy ligand.

The computed Os-N bond lengths of the bis(terpyridine)osmium(II) complex (**1**) vary from 2.009 (central bonds) to 2.102 (terminal bonds) Å, which is up to 0.05 Å longer than the Os-N experimental bond lengths for complex (**1**) that vary from 1.964 (central bonds) to 2.083 (terminal bonds) Å. The longer Os-N bond lengths obtained from calculations in comparison with X-ray crystallography results are anticipated, as “chemical pressure” within the solid-state crystal structure tends to shorten metal–ligand bond lengths beyond what is predicted by gas-phase or implicit solvent models. The slightly longer calculated bond lengths, along with the low AD and MAD values presented in Table 4, indicate that the chosen DFT approach is well suited for accurately characterizing the geometry of the bis(terpyridine)osmium(II) complexes in this work.

#### 2.3.2. Ground State for Oxidized and Reduced Bis(terpyridine)osmium(II)

Table 5 provides results that confirm the effectiveness of the selected DFT approach in correctly identifying the electronic structure, energy levels, and ground states of [Os(tpy)_2_]^m^. The data show that the ground states, or lowest energy configurations, for m = 0, 1, 2, and 3 correspond to S = 1 (triplet), ½ (doublet), 0 (singlet), and ½ (doublet), respectively. [Os(tpy)₂]^2+^ is diamagnetic, and the NMR data confirm that it has a singlet ground state.

#### 2.3.3. Electronic Structure of Bis(terpyridine)osmium(II)

**Figure 4:**
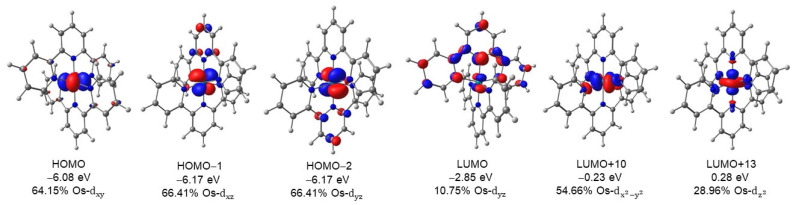
The Os d-based orbitals and the LUMO of the B3LYP/6-311G(d,p)/def2tzvpp-computed [Os(tpy)_2_]^2+^. The energy and % Os-d character of the MOs are indicated. Contour = 0.06 Åe^−3^.

In this division, the electronic structure for the bis(terpyridine)osmium(II) complex is discussed, with a focus on the molecular orbitals (MOs) that are obtained from DFT computations. Examining the nature and energy of these orbitals provides important information about the molecular redox processes that are observed experimentally. The singlet bis(terpyridine)osmium(II), (**1**), is a d^6^ species with spin state S = 0. This pseudo-octahedral molecule thus has six electrons in the lower-energy t_2g_ ($d_{\mathrm{xy}}$, $d_{\mathrm{yz}}$ and $d_{\mathrm{xz}}$ orbitals) set and no electrons in the higher-energy e_g_ ($d_{x^{2}-y^{2}}$ and $d_{z^{2}}$ orbitals) set. Figure 4 shows that the mainly d-based MOs of this d^6^ complex with S = 0 are given by $d_{\mathrm{xy}}^{2}d_{\mathrm{xz}}^{2}d_{\mathrm{yz}}^{2}$.

**Figure 5:**
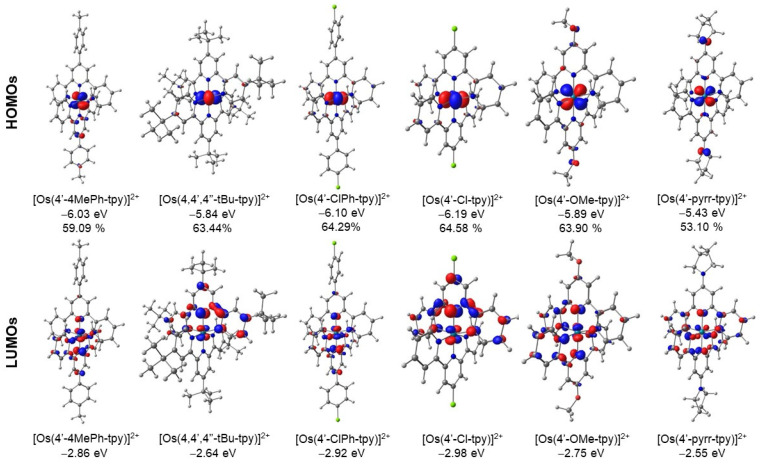
The HOMO and LUMO of the B3LYP/6-311G(d,p)/def2tzvpp-computed (**2**)–(**7**). The energy and % Os-d character of the HOMO are indicated. Contour used for MO plots = 0.06 Åe^−3^.

Similarly, as shown in Figure 4 for (**1**), the highest occupied MOs (HOMOs) of compounds (**2**) through (**7**) are mostly found on osmium (Figure 5), suggesting that the electron removal process involved in oxidation, or Os(II/III) oxidation, will be metal-centric. On the other hand, as for compound (**1**) shown in Figure 4, the lowest unoccupied MOs (LUMOs) of (**2**) through (**7**) are located mainly on the aromatic backbone of the terpyridine ligands (Figure 5). The aromatic backbone of the terpyridine ligands is the site of the reduction process of (**1**)–(**7**), which involves the uptake of an electron into the LUMO.

#### 2.3.4. Bis(tepyridine)osmium(III)

**Scheme 5:**
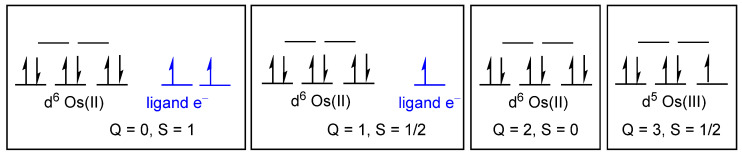
Illustration of the electron configuration for metal-d orbitals (with e_g_ on top and t_2g_ on the bottom, shown in black) and unpaired ligand electrons (depicted in blue) of distorted octahedral bis(terpyridine)osmium(II) (Q = 2), along with its oxidized (Q = 3) and reduced (Q = 1 and Q = 0) forms.

Bis(terpyridine)osmium(III) is produced when bis(terpyridine)osmium(II) is oxidized. The d^5^ bis(terpyridine)osmium(III) complex has a spin state of S = 1⁄2 (Table 5). Scheme 5 illustrates that following oxidation, the molecule has five electrons (one unpaired α-electron) in the t_2g_ orbital set and no electrons in the higher energy e_g_ orbital set. In d^5^ bis(terpyridine)osmium(III), weak Jahn–Teller distortion is possible, because of the degeneracy of the t_2g_ orbital set [49]. However, Jahn–Teller distortion is noticeable when degeneracy of the e_g_ orbitals ($d_{x^{2}-y^{2}}$ and $d_{z^{2}}$) occurs, since these orbitals are aligned along the coordinate system axes in the direction of the ligands, causing a substantial energy gain from Jahn–Teller distortion. Jahn–Teller distortion with degeneracy of the t_2g_ orbitals is much weaker, since the t_2g_ orbitals do not point directly towards the atoms of the coordinating ligands, causing a smaller energy gain.

**Figure 6:**
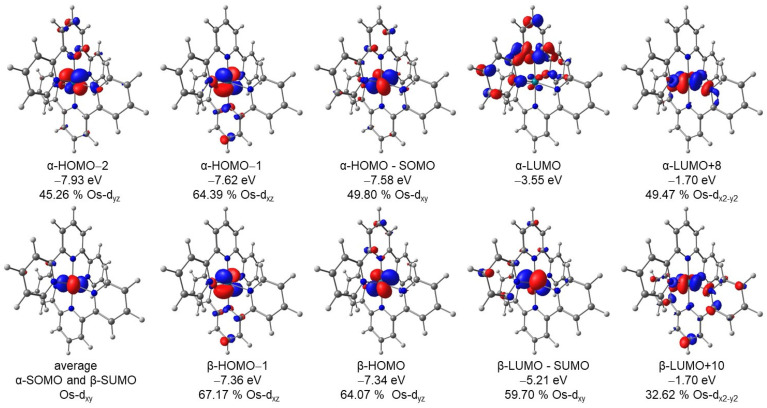
The B3LYP/6-311G(d,p)/def2tzvpp CH_3_CN-computed MOs and spin density plot of the [Os(tpy)_2_]^3+^ doublet. The energy and % Os-d character of the MOs are indicated. Contour = 0.06 (0.006) Åe^−3^ for the MO (spin) plots.

**Figure 7:**
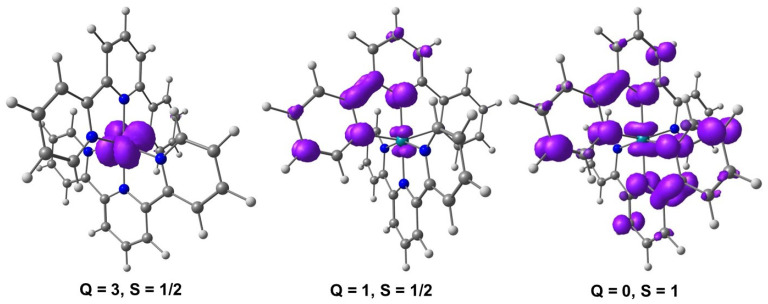
Spin density plots (contour = 0.006 Åe^−3^) of the B3LYP/6-311G(d,p)/def2tzvpp CH_3_CN-computed geometries of [Os(tpy)_2_]^m^, with m = 3, 1, and 0.

As obtained for bis(terpyridine)osmium(II), the four terminal Os-N bonds in bis(terpyridine)osmium(III) are longer than the two central bonds due to the strain in the tpy ligand. Os(III) has a higher positive charge than Os(II), which makes it more electron-deficient. Shorter Os-N bond lengths for Os(III) are thus expected as a result of stronger electrostatic interactions between osmium and the N atoms. The Os-N_terminal_ bonds of Os(III) are very similar to that of Os(II), while the Os-N_central_ bonds of Os(III) show a slight increase in length (Table 6), compared to Os(II), which could be a consequence of the Jahn–Teller effect. The singly occupied MO (SOMO; α-HOMO) and the singly unoccupied MO (SUMO; β-LUMO) of bis(terpyridine)osmium(III) are of Os-d_xy_ character (z-axis defined along the N_central_-Os-N_central_ direction), as is clear from the MO presentation of the average of the SOMO and SUMO, suggesting a small elongation Jahn–Teller effect along the N_central_-Os-N_central_ direction (Figure 6). The bis(terpyridine)osmium(III) spin density plot is displayed in Figure 7. The character of the spin plot agrees with that of the SOMO in the t_2g_ orbital set, namely, of primarily Os-d_xy_ character (z-axis defined along the N_central_-Os-N_central_ direction).

#### 2.3.5. Reduced and Doubly Reduced Bis(terpyridine)osmium(II)

[Os(tpy)_2_]^1+^ with spin S = ½ is obtained upon the first one-electron reduction of bis(terpyridine)osmium(II). An analysis of the Mulliken spin density of [Os(tpy)_2_]^1+^ reveals that the Mulliken spin on Os is only 0.09 e^−^, while on one of the tpy ligands, it is 0.86 e^−^ and on the other tpy ligand, it is 0.05 e^−^, see values in Table 7. These values, together with the spin density plot of [Os(tpy)_2_]^1+^ in Figure 7, make it evident that this [Os(tpy)_2_]^1+^ molecule is composed of one neutral tpy ligand, one tpy radical (which contains one unpaired electron), and an Os(II) center (which contains no unpaired electrons), formulated as [Os(tpy)(tpy^•^)]^1+^, as depicted in Scheme 5. This formulation is consistent with the description of [Os(tpy)_2_]^1+^ in Section 2.2 (CV results), that reduction occurs on the tpy ligand.

Os(tpy)_2_]^0^ with spin S = 1 is obtained upon the second one-electron reduction of bis(terpyridine)osmium(II). An analysis of the Mulliken spin density of [Os(tpy)_2_]^0^ reveals that the Mulliken spin on Os is only 0.22 e^−^, while on both of the tpy ligands, it is 0.89 e^−^ each, see values in Table 7. These values, together with the spin density plot of [Os(tpy)_2_]^0^ shown in Figure 7, make it evident that [Os(tpy)_2_]^0^ is composed of two tpy radicals (each of which contains one unpaired electron), and an Os(II) center (which contains no unpaired electrons), formulated as [Os(tpy^•^)_2_]^0^, as illustrated in Scheme 5.

**Scheme 6:**
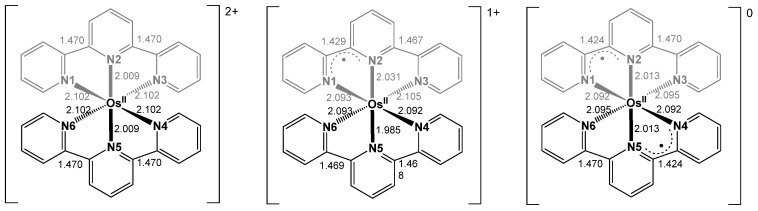
Selected bond lengths (Å) of B3LYP/6-311G(d,p)/def2tzvpp CH_3_CN-computed geometries of [Os(tpy)_2_]^2+^, [Os(tpy)(tpy^•^)]^1+^ and [Os(tpy^•^)_2_]^0^. The two flat terpyridine ligands are orientated perpendicular to each other. The bonds at the metal center of the distorted octahedron are represented as follows: The bonds extending towards the front are depicted as solid triangular wedge shapes, while the bonds extending to the back are illustrated using dashed lines. The metal-N bonds that lie in the plane of the paper are shown with broad solid lines [49].

Selected bond lengths of [Os(tpy)_2_]^m^*,* m = 0–3, are given in Table 6 and depicted in Scheme 6 for m = 0–2. While the bond lengths of both terpyridine ligands in the m = 2 geometry are equivalent, they differ for the m = 1 optimized geometry. In the m = 1 geometry, one tpy is a (tpy^•^)^1–^ π-radical anion and the other tpy is a neutral tpy^0^ ligand. The (tpy^•^)^1–^ π-radical is non-symmetrically coordinated to the Os(II) ion having one short C_pyridine_-C_pyridine_ distance of 1.429 Å, characteristic of a (tpy^•^)^1–^ π-radical [50], and a second longer C_pyridine_-C_pyridine_ of 1.467 Å, which is more common of a neutral ligand. The C_pyridine_-C_pyridine_ bond length of the neutral tpy ligands in [Os(tpy)_2_]^2+^ is 1.470 Å. Analysis of the Mulliken spin density of the m = 1 geometry indicates that the unpaired electron on the (tpy^•^)^1–^ π-radical is mainly located on the two pyridines that are connected by the shorter C_pyridine_-C_pyridine_ bond. The m = 0 geometry contains two identical (tpy^•^)^1–^ π-radicals (Scheme 6). Both (tpy^•^)^1–^ π-radicals are non-symmetrically coordinated to Os(II), with the spin density in each radical mainly located on the two pyridines that are connected by the shorter C_pyridine_-C_pyridine_ bond, see spin plot in Figure 7.

For both reduced geometries (m = 1 and m = 0), there is only a small change in the Os-N bonds (values in Table 4) and in the electronic structure (no significant re-arrangement of the d-MO levels upon reduction, see Scheme 5), consistent with a central Os(II) center and reversible electrochemical behavior upon reduction of [Os(tpy)_2_]^2+^. The quasi-reversible electrochemical behavior experimentally observed for the second reduction may result from the instability of the ligand-based radicals that formed during the reduction (Figure 7).

### 2.4. Combining Experimental Results with Theoretically Calculated Values and Electronic Parameters

This section discusses how the experimental oxidation and reduction potentials of bis(terpyridine)osmium(II) complexes (**1**) through (**7**) are related to DFT-calculated energies and charges and Hammett substituent constants. The available oxidation potentials from [28] of complex (**8**)–(**15**) are added to broaden the range of oxidation values. Since the reduction of complexes (**8**)–(**15**) was reported to be irreversible, their values cannot be used in relationships [28]. The experimental oxidation potentials, DFT-computed solvent phase (CH_3_CN) energies for (**1**)–(**15**), and Hammett substituent constants for complexes (**1**)–(**7**) are given in Table 8. The DFT solvent phase (CH_3_CN)-computed NBO charges (Q in e^−^), MESP charge (Q in e^−^), and potential (V in au) for (**1**)–(**15**) are provided in Table 9.

#### 2.4.1. Hammett Constants

**Figure 8:**
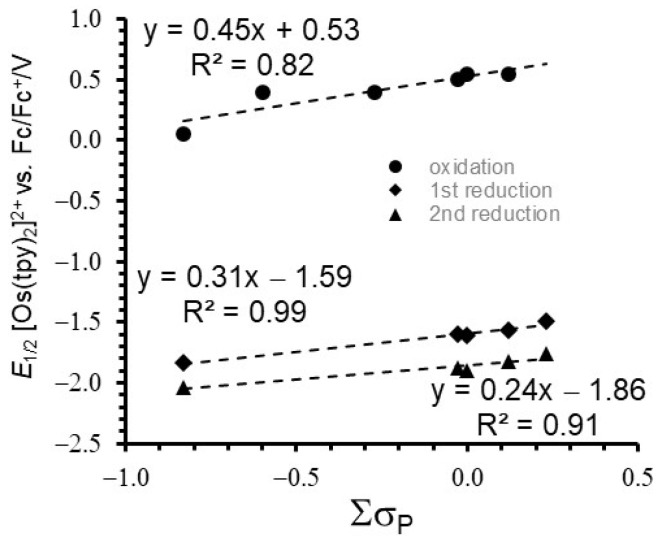
Relationship between the experimental oxidation and reduction potentials of (**1**)–(**7**) and the sum of the Hammett substituent parameters of the substituents on the tpy-ligands.

In the scientific literature, the Hammett substituent constants (σ) [51] are widely used to quantify the electronic effect on the central metal (oxidation) and the ligands (reduction) of a substituent group on a phenyl or a related aromatic group in the complex to which it is bound [52]. The oxidation potential (*E*_1/2,ox_) and the two reduction potentials are correlated with the sum of σ of the substituents on the tpy-ligand in compounds (**1**) to (**7**), see Figure 8. The strong correlation across all three trends indicates that σ can be used as a measure of the electronic effect of the substituent groups in [Os(tpy)_2_]^2+^ complexes containing differently substituted tpy ligands.

#### 2.4.2. DFT Energies

**Figure 9:**
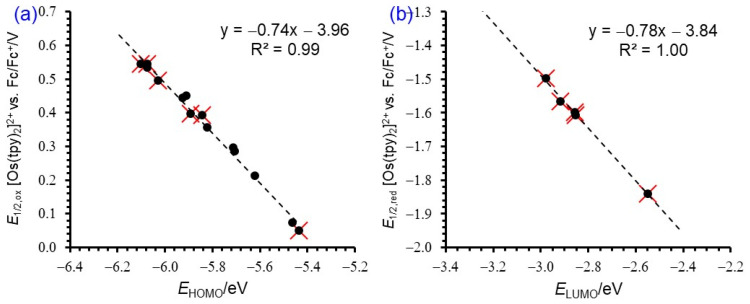
Relationship between the experimental (**a**) *E*_1/2,ox_ and DFT solvent phase (CH_3_CN)-calculated *E*_HOMO_ and (**b**) *E*_1/2,red_ and the DFT solvent phase (CH_3_CN)-calculated *E*_LUMO_, for the series of [Os(tpy)_2_]^2+^ (this study, data highlighted with red crosses), [Os(bpy)_3_]^2+^ and [Os(phen)_3_]^2+^ (from the literature).

**Figure 10:**
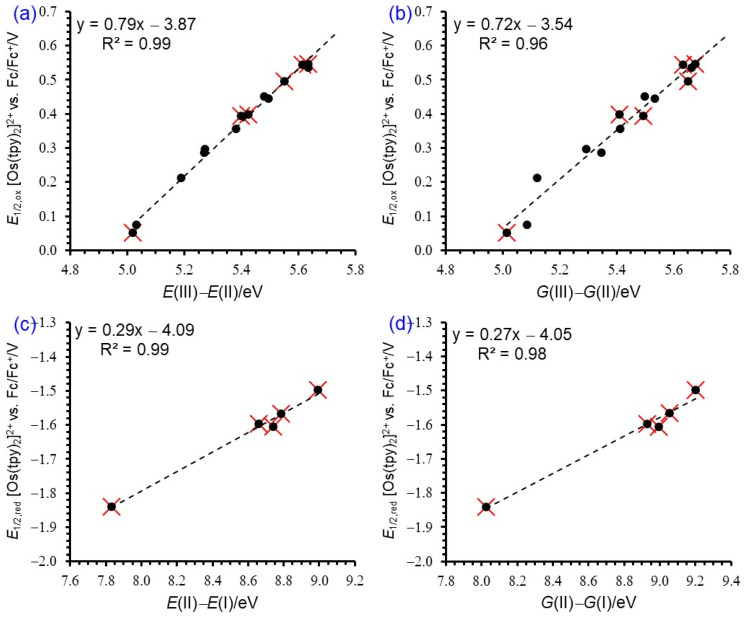
Relationship between the experimental *E*_1/2,ox/red_ and (**a**,**c**) the DFT-calculated electronic energy *E* difference and (**b**,**d**) the DFT-calculated free energy *G* difference of the DFT solvent phase (CH_3_CN) optimized [Os(tpy)_2_]^2+^ (this study, data highlighted with red crosses), [Os(bpy)_3_]^2+^ and [Os(phen)_3_]^2+^ (from the literature) and their oxidized/reduced forms.

As detailed in Section 2.3 (Theoretical calculations), oxidizing a molecule involves removing an electron from the HOMO, whereas reducing a molecule requires gaining an electron in the LUMO. Therefore, the values of both oxidation and reduction processes are directly linked to the energy levels of the HOMO and LUMO, respectively. This correlation is depicted in Figure 9 for the oxidation of (**1**)–(**15**) and the reduction of complexes (**1**), (**2**), (**4**), (**5**), and (**6**). The solvent-phase-calculated electronic (*E*) and free (*G*) energy difference between bis(terpyridine)osmium(II) and its oxidized or reduced forms is related to the oxidation/reduction potential (removing an electron from/adding an electron to the molecule in the solvent phase) of bis(terpyridine)osmium(II), see Figure 10.

**Figure 11:**
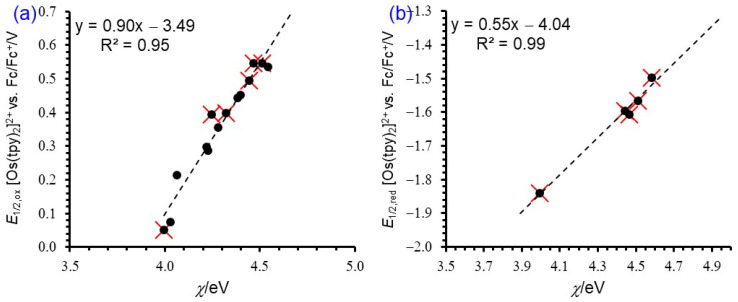
Relationship between the experimental (**a**) *E*_1/2,ox_ and (**b**) *E*_1/2/,red_ and the DFT solvent phase (CH_3_CN)-calculated electronegativity (*χ*) of the molecules, for the series of [Os(tpy)_2_]^2+^ (this study, data highlighted with red crosses), [Os(bpy)_3_]^2+^ and [Os(phen)_3_]^2+^ (from the literature).

**Figure 12:**
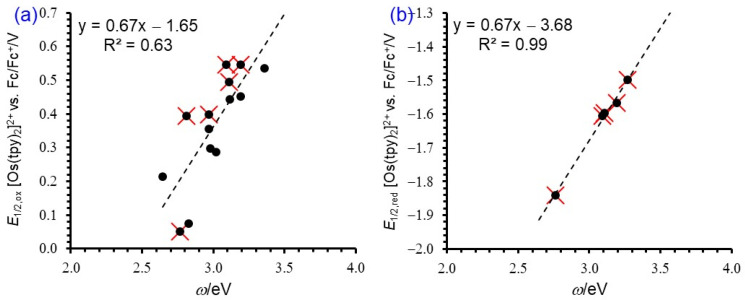
Relationship between the experimental (**a**) *E*_1/2,ox_ and (**b**) *E*_1/2,red_ and the DFT solvent phase (CH_3_CN)-calculated electrophilicity index (*ω*) of the molecules, for the series of [Os(tpy)_2_]^2+^ (this study, data highlighted with red crosses), [Os(bpy)_3_]^2+^ and [Os(phen)_3_]^2+^ (from the literature).

DFT calculations provide global reactivity parameters that describe the overall behavior of molecules, such as electronegativity (*χ*) and the electrophilicity index (*ω*) [53]. Electronegativity, which stays constant throughout an atom or molecule and does not vary between orbitals [54], reflects the inclination of an atom or molecule to attract electrons [55]. This property influences the electronic characteristics of both the metal and ligands in a complex. In bis(terpyridine)osmium(II) complexes, lower *χ* values are typically found when the tpy ligands have electron-donating groups, such as 4′-pyrr-tpy, which is associated with a lower Hammett *para* substituent parameter (σ_p_, data in Table 8, relationship in Figure 11). Similarly, the global electrophilicity index (*ω*) measures the electrophilic nature of atoms and molecules [56]. Higher *ω* values indicate a greater reactivity toward electrophiles. Like *χ*, bis(terpyridine)osmium(II) complexes with electron-donating substituents on the tpy ligands, such as 4′-pyrr-tpy, exhibit lower *ω* values, as reflected in the lower *para* Hammett parameter (data in Table 8, relationship shown in Figure 12). Both the oxidation potential *E*_1/2,ox_, and the reduction potential *E*_1/2,red_, are directly proportional to the electronegativity (*χ*) and the electrophilicity index (*ω*), as shown in Figure 11 and Figure 12, respectively.

#### 2.4.3. DFT Charges and Potentials

Electronic density, charges, and potentials are examples of local reactivity parameters whose values depend on where they exist in the molecule [53]. The characterization of site-specific reactivity patterns is made possible by this feature.

**Figure 13:**
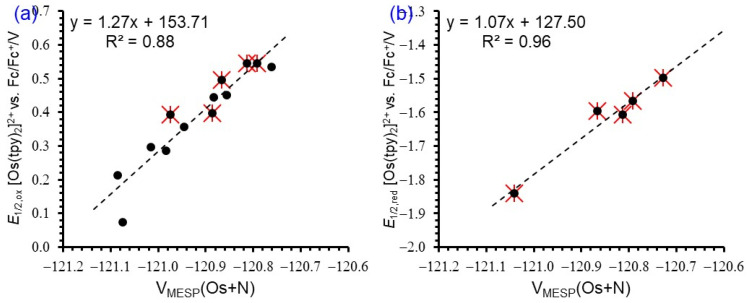
Relationship between the experimental (**a**) *E*_ox_ and (**b**) *E*_red_ and the DFT solvent phase (CH_3_CN)-calculated MESP (au), for the series of [Os(tpy)_2_]^2+^ (this study, data highlighted with red crosses), [Os(bpy)_3_]^2+^ and [Os(phen)_3_]^2+^ (from the literature). Data of (**7**) did not fit in (**a**).

The molecular electrostatic potential (MESP) provides a visual representation of the electrostatic potential energy distribution around a molecule. It offers valuable information about the molecule’s charge distribution. The MESP on osmium and the coordinated nitrogens is utilized to measure the electronic impact of different tpy substituents on the OsN_6_ core in (**1**)–(**15**). MESP is frequently used in the literature to forecast compounds’ redox potential [57]. As shown in Figure 13, the MESP on the OsN_6_ core is related to both oxidation and reduction potentials.

**Figure 14:**
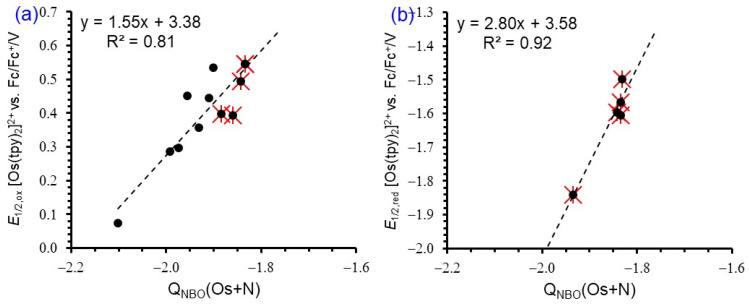
Relationship between the experimental (**a**) *E*_ox_ and (**b**) *E*_red_ and the DFT solvent phase (CH_3_CN)-calculated NBO charges Q (e^−^), for the series of [Os(tpy)_2_]^2+^ (this study, data highlighted with red crosses), [Os(bpy)_3_]^2+^ and [Os(phen)_3_]^2+^ (from the literature). Data of (**7**) and (**15**) did not fit in (**a**).

Natural Bond Orbital (NBO) charges provide insight into the distribution of electron density within a molecule. Specifically, NBO charges quantify the partial charge on individual atoms, indicating whether an atom is electron-rich (negative charge) or electron-deficient (positive charge). They highlight how differences in electronegativity between atoms affect charge distribution in a molecule. Lower (less positive and more negative) NBO charges, as seen in Figure 14, indicate a higher electron density surrounding the OsN_6_ core, making it easier to remove electrons from the molecule and lowering the oxidation potential.

## 3. Materials and Methods

### 3.1. General

Melting points (m.p.s) were determined with the differential scanning calorimetry DSC 2500 discovery series. The data were analyzed on the TRIOS software version 5.1. UV/Vis measurements were recorded on a Shimadzu UV-1800PC UV spectrophotometer (Shimadzu, Kyoto, Japan), equipped with a multi-cell thermostatted cell holder (±0.1 °C). FTIR measurements (solid samples, 16 scans per sample with a resolution of 0.5 cm^−1^) were determined with a Nicolet iS50 FTIR Spectrometer ATR running Omnic software version 9.15. Nuclear Magnetic Resonance spectroscopy**:** The liquid-state ^1^H and ^13^C NMR spectra were recorded at 25.0 °C on a 400 MHz Avance II Bruker spectrometer (Bruker, Johannesburg, South Africa) operating at 400.13 and 100.61 MHz for ^1^H and ^13^C, respectively. Deuterated acetone was used as solvent. The chemical shifts (δ) are reported in parts per million (ppm), and the spectra are referenced relative to Me_4_Si internal standard at 0 ppm. Coupling constants (*J*) are reported in Hz. Powder X-ray diffraction (PXRD): A Malvern-Panalytical Empyrean X-ray diffraction (PXRD) instrument with a Cu radiation source (45 kV and 40 mA) and an X’Celerator detector was used. Samples were analyzed on a zero-background sample holder. The samples are run from 3.5 to 70 deg, step 0.008 deg and 99 s per step. UV/Vis, FTIR, PXRD, and NMR spectra are provided in the Supplementary Materials.

### 3.2. Synthesis of Complexes *(**1**)*–*(**7**)*

Sigma-Aldrich supplied solvents, and synthesis chemicals were employed without additional purification. Osmium complexes were synthesized following established methods in the literature with minor adjustments [28]. Dried OsCl_3_ (0.1488 g, 0.502 mmol) was dissolved in ethylene glycol (20 mL) and deionized water (2 mL). The solution was refluxed until the metal salt was dissolved, for 15–30 min, obtaining a dark green solution. Terpyridine (0.2327 g, 0.998 mmol) was added resulting in a brown solution. Ascorbic acid (0.0893 g, 0.507 mmol) was added, and the solution refluxed for another 20 min at 150 °C, the brown color changing to dark brown. After cooling, the solution was diluted to 40 mL and the pH adjusted to 8 by the addition of a few drops of NaOH solution (2.5 M). NaBF_4_ (2.0835 g, 18.98 mmol) was added and the solution cooled on ice. After vacuum filtration, washing with cold water, and drying, 0.1950 g [Os(2,2′:6′,2′′-terpyridine)_2_](BF_4_)_2_ product was obtained. Characterization data of (**1**)–(**7**) are provided below.

#### 3.2.1. [Os (2,2′:6′,2′′-terpyridine)_2_](BF_4_)_2_ (**1**)

Yield: 46.80%; Color: Dark brown; M.p.: 108.99 °C; UV: λ (nm) (ε (mol^−1^dm^3^cm^−1^)) 655 (2105), 476 (8,438), 311 (45,618), 270 (31,859), 229 (40,169) (CH_3_CN); (lit for [Os (tpy)_2_](Cl)_2_ in ethanol-methanol (4/1:*v*/*v*) λ (ε): 657 (3650), 477 (13,750), 312 (66,250), 271 (38,850), 227 (37,900) [37]). ν (cm^−1^) 3059 (C-H), 1597 (C=C); ^1^H NMR: (400 MHz, (CD_3_)_2_CO, 25 °C): δ 7.24–7.27 (4H, m, CH), 7.59 (4H, d, ^3^*J* = 5.6 Hz, CH), 7.91–7.95 (4H, m, CH), 8.06–8.10 (2H, m, CH), 8.80 (4H, d, ^3^*J* = 8.0 Hz, CH), 9.09 (4H, d, ^3^*J* = 8.4 Hz, CH).

#### 3.2.2. [Os (4′-(4-methylphenyl)-2,2′:6′,2′′-terpyridine)_2_](BF_4_)_2_ (**2**)

Yield: 64.48%; Color: Dark maroon; M.p.: 168.12 °C; UV: λ (nm) (ε (mol^−1^dm^3^cm^−1^)) 665 (1728), 490 (7853), 316 (42,637), 286 (42,288), 228 (40,674) (CH_3_CN); (lit for [Os(4MePh-tpy)_2_](PF_6_)_2_ in ethanol-methanol (4/1:*v*/*v*) λ (ε): 668 (7700), 490 (29,750), 315 (83,900), 286 (64,900), 202 (98,600) [37]). ν (cm^−1^) 3033 (C-H), 1598 (C=C); ^1^H NMR: (400 MHz, (CD_3_)_2_CO, 25 °C): δ 2.57 (6H, s, CH), 7.28–7.31 (4H, m, CH), 7.59 ( 4H, d, ^3^*J* = 8.0 Hz, CH), 7.72 (4H, d, ^3^*J* = 5.6 Hz, CH), 7.96–8.00 (4H, m, CH), 8.23 (4H, d, ^3^*J* = 8.0 Hz, CH), 9.07 (4H, d, ^3^*J* = 8.4 Hz, CH), 9.46 (4H, s, CH).

#### 3.2.3. [Os (4,4′,4”-tri-tert-Butyl-2,2′:6′,2′′-terpyridine)_2_](BF_4_)_2_ (**3**)

Yield: 65.54%; Color: Maroon; M.p.: 219.46 °C; UV: λ_max_ 238 nm, ε_max_ 48,631 mol^−1^dm^3^cm^−1^ (CH_3_CN). ν (cm^−1^) 2956 (C-H), 1586 (C=C); ^1^H NMR: (400 MHz, (CD_3_)_2_CO, 25 °C): δ 1.45–1.47 (54H, m, CH), 7.49 (4H, s, CH), 8.59–8.63 (8H, m, CH), 8.81 (4H, s, CH).

#### 3.2.4. [Os (4′-(4-chlorophenyl)-2,2′:6′,2′′-terpyridine)_2_](BF_4_)_2_ (**4**)

Yield: 56.34%; Color: Dark maroon; M.p.: 173.05 °C; UV: λ_max_ 285 nm, ε_max_ 10,340 mol^−1^dm^3^cm^−1^ (CH_3_CN). ν (cm^−1^) 3065 (C-H), 1600 (C=C); ^1^H NMR: (400 MHz, (CD_3_)_2_CO, 7.28–7.31 (4H, m, CH), 7.72 (4H, d, ^3^*J* = 5.2 Hz, CH), 7.80 (4H, d, ^3^*J* = 8.4 Hz, CH), 7.96–8.02 (4H, m, CH), 8.34 (4H, d, ^3^*J* = 8.0 Hz, CH), 9.06 (4H, d, ^3^*J* = 8.4 Hz, CH), 9.51 (4H, s, CH).

#### 3.2.5. [Os (4′-chloro-2,2′:6′,2′′-terpyridine)_2_](BF_4_)_2_ (**5**)

Yield: 67.86%: Color: Dark purple; M.p.: 153.80 °C; UV: λ_max_ 240 nm, ε_max_ 45,805 mol^−1^dm^3^cm^−1^ (CH_3_CN). ν (cm^−1^) 3086 (C-H), 1555 (C=C); ^1^H NMR: (400 MHz, (CD_3_)_2_CO, 25 °C): δ 7.50–7.52 (4H, m, CH), 8.00–8.04 (4H, m, CH), 8.52 (4H, s, CH), 8.69–8.74 (8H, m, CH).

#### 3.2.6. [Os (4′-methoxy)-2,2′:6′,2′′-terpyridine)_2_](BF_4_)_2_ (**6**)

Yield: 24.19%; Color: Dark purple; M.p.: 289.28 °C; UV: λ_max_ 236 nm, ε_max_ 44,762 mol^−1^dm^3^cm^−1^ (CH_3_CN). ν (cm^−1^) 3010 (C-H), 1587 (C=C); ^1^H NMR: (400 MHz, (CD_3_)_2_CO, 25 °C): δ 4.06 (6H, s, CH), 7.45 (4H, s, CH), 7.95–7.99 (4H, m, CH), 8.10 (4H, s, CH), 8.69 (8H, d, ^3^*J* = 8.0 Hz, CH).

#### 3.2.7. [Os 4′-(N-Pyrrolidinyl)-2,2′:6′,2′′-terpyridine)_2_](BF_4_)_2_ (**7**)

Yield: 38.64%; Color: Dark purple; M.p.: 251.24 °C; UV: λ_max_ 282 nm, ε_max_ 14,539 mol^−1^dm^3^cm^−1^ (CH_3_CN). ν (cm^−1^) 3069 (C-H), 1610 (C=C); ^1^H NMR: (400 MHz, (CD_3_)_2_CO, 25 °C): δ 2.20–2.23 (8H, m, CH), 3.87 (8H, s, CH), 7.71–7.74 (4H, m, CH), 7.77 (4H, s, CH), 8.14–8.18 (4H, m, CH), 8.62 (4H, d, ^3^*J* = 8.0 Hz, CH), 8.90 (4H, d, ^3^*J* = 4.0 Hz, CH).

### 3.3. Cyclic Voltammetry

Cyclic voltametric (CV) measurements were conducted on a BAS100B Electrochemical Analyzer linked to a personal computer, utilizing the BAS100W Version 2.3 software. Measurements were performed at 293 K, and temperature was kept constant to within 0.5 K. A three-electrode cell was used, with a glassy carbon (surface area 1.257 × 10^−5^ m^2^) working electrode, Pt auxiliary electrode and a Ag/Ag^+^ (0.010 mol dm^−3^ AgNO_3_ in CH_3_CN) reference electrode, mounted on a Luggin capillary, as described and referenced in our previous work [28]. The working electrode was polished on a Bühler polishing mat, first with 1 micron and then with ¼ micron diamond paste (in a figure-of-eight motion), rinsed with EtOH, H_2_O and CH_3_CN, and dried before each experiment. The electrochemistry measurements were performed in solvent CH_3_CN, containing 0.1 mol dm^−3^ tetrabutylammonium hexafluorophosphate (TBAPF_6_) as supporting electrolyte. The voltammograms were obtained at room temperature under a blanket of argon. The concentration of the different samples was ca 0.003 mol dm^−3^. Scan rates (ν) were 0.02–5.00 V s^−1^. Ferrocene was used as an internal standard, and cited potentials were referenced against the Fc/Fc^+^ couple, as suggested by IUPAC. *E*_pa_ = peak anodic potential and *i_pa_* = peak anodic current; *E*_pc_ = peak cathodic potential and *i_pc_* = peak cathodic current. The reduction potential is determined by the mean of the oxidation and reduction potential *E_1/2_* = (*E*_pa_ − *E*_pc_)/2, and the peak potential separation Δ*E*_p_ = *E*_pa_ − *E*_pc_.

### 3.4. DFT Methods

Density functional theory (DFT) calculations were performed on the neutral, reduced, and oxidized molecules using the B3LYP functional which is composed of the Becke 88 exchange functional [58] in combination with the LYP correlation functional [59], as implemented in the Gaussian 16 package [60]. The triple-ζ basis set 6-311G(d,p) was used for lighter atoms (C, H, F, O) and the def2-TZVPP basis set for both the core and valence electrons of Os. Optimizations were performed using CH_3_CN as a solvent, since the reported experimental redox potentials were obtained in CH_3_CN solution. The implicit solvent Polarizable Continuum Model (PCM) [61] that uses the integral equation formalism variant (IEFPCM) [62] was used for solvent calculations in Gaussian. Frequency calculations were performed on all DFT-optimized molecules to confirm that no local minima were mistakenly identified, as indicated by the absence of imaginary frequencies. The input coordinates for the compounds were constructed using Chemcraft [63].

The following formula, as described and referenced in our previous work, was used to calculate DFT energies, potentials, and charges [64]. According to the Koopmans’ theorem, the ionization potential (IP) can be approximated by the negative of the HOMO energy (and similarly, the LUMO energies provide an approximation to electron affinity, EA):

IP = −*E*_HOMO_

EA = −*E*_LUMO_.

DFT energies, global electrophilicity index (ω), Mulliken electronegativity (χ), chemical hardness (*η*), and chemical potential (*μ*) were calculated using the following formulae:

χ = (IP + EA)/2

*μ* = −(IP + EA)/2 = −χ

*η* = IP − EA

ω = (*μ*^2^/2*η*) = ((IP + EA)/2)^2^/(2 × (IP − EA)) = (IP + EA)^2^/(6 × (IP − EA))

NBO charges were obtained from the NBO calculation in Gaussian 16. The molecular electrostatic potential (MESP) was obtained from the electrostatic potential calculation in Gaussian 16 program. The MESP parameters were calculated from the standard equation for the potential V(*r*) in atomic units (Hartree, 1 Hartree = 627.503 kcal/mol = 27.2114 eV = 2 625.5 kJ/mol), at a point *r* expressed as follows:

$$V\left(r\right)=\overset{N_{A}}{\underset{A}{\sum }}\frac{Z_{A}}{\left|r-R_{A}\right|}-\int \frac{\rho \left(r^{\prime }\right)d^{3}r^{\prime }}{\left|r-r^{\prime }\right|}$$

The *ρ*(*r*′) in the expression is the electron density, *N*_A_ is the total number of nuclei, and *Z*_A_ in the charge on nucleus A at position *R*_A_.

The MO and spin plots were created from the DFT output files and visualized using Chemcraft [63]. The color scheme for atoms (online version) was as follows: Os (turquoise), N (blue), C (grey), O (red), Cl (green), and H (off-white).

## 4. Conclusions

The oxidation and reduction of bis(terpyridine)osmium(II) complexes are based on the osmium and terpyridine ligands, respectively, as confirmed by DFT calculations and supported by the literature. The measured redox potentials and Hammett constants, as well as the DFT-calculated energies associated with the particular redox process, are found to have linear relationships due to the substituents’ donating/withdrawing nature, which is measured by Hammett constants. This causes the experimental redox potentials to shift to lower or higher values.

## Figures and Tables

**Table 1:** Electrochemical data (potential in V vs. Fc/Fc^+^) measured in CH_3_CN of the oxidation of bis(terpyridine)Os(II) compounds from this work (tw) at ν = 0.100 Vs^−1^ and published works.

No	Ligand	Electrolyte ^a^	Ref Electrode ^b^	*E*_1/2_ (Reported)	*E*_1/2_ (Fc/Fc^+^)	∆*E*	*E*_pa_	Ref
1	tpy	TBAPF_6_	Fc/Fc^+^		0.546	0.068	0.580	tw
	tpy	TEAP	SCE	0.970	0.554			[35]
	tpy	TEABF_4_	SCE	0.960	0.544			[36]
	tpy	TEABF_4_	SCE	0.970	0.554			[37]
2	4′-4MePh-tpy	TBAPF_6_	Fc/Fc^+^		0.495	0.089	0.539	tw
	4′-4MePh-tpy	TEABF_4_	SCE	0.890	0.474			[19]
	4′-4MePh-tpy	TEABF_4_	SCE	0.930	0.514			[36]
	4′-4MePh-tpy	TEABF_4_	SCE	0.930	0.514			[37]
3	4,4′,4”-tBu-tpy	TBAPF_6_	Fc/Fc^+^		0.394	0.087	0.437	tw
4	4′-ClPh-tpy	TBAPF_6_	Fc/Fc^+^		0.545	0.066	0.578	tw
6	4′-OMe-tpy	TBAPF_6_	Fc/Fc^+^		0.398	0.060	0.428	tw
7	4′-pyrr-tpy	TBAPF_6_	Fc/Fc^+^		0.051	0.059	0.080	tw

^a^ TBAPF_6_ = tetrabutylammonium hexafluorophosphate, TEAP = Tetraethylammonium perchlorate, TEABF_4_ = tetrabutylammonium tetrafluoroborate. ^b^ Converting potential *E* vs. SCE to vs. Fc/Fc^+^, 0.416 V was used (*E*(Fc/Fc^+^) = 0.66(5) V vs. NHE in TBAPF_6_/CH_3_CN [42] and SCE = 0.244 V vs. NHE).

**Table 2:** Electrochemical data (potential in V vs. Fc/Fc^+^) measured in CH_3_CN of the 1st reduction of bis(terpyridine)Os(II) compounds from this work (tw) at ν = 0.100 Vs^−1^ and published works.

No	Ligand	Electrolyte ^a^	Ref Electrode ^b^	1st Reduction				Ref
				*E*_1/2_ (Reported)	*E*_1/2_ (Fc/Fc^+^)	∆*E*	*E*_pc_	tw
1	tpy	TBAPF_6_	Fc/Fc^+^		−1.606	0.070	−1.641	tw
	tpy	TEAP	SCE	−1.25	−1.666			[35]
	tpy	TEABF_4_	SCE	−1.25	−1.67			[36]
	tpy	TEABF_4_	SCE	−1.23	−1.65			[37]
2	4′-4MePh-tpy	TBAPF_6_	Fc/Fc^+^		−1.597	0.078	−1.636	tw
	4′-4MePh-tpy	TEABF_4_	SCE	−1.17	−1.59			[19]
	4′-4MePh-tpy	TEABF_4_	SCE	−1.23	−1.65			[36]
	4′-4MePh-tpy	TEABF_4_	SCE	−1.22	−1.64			[37]
4	4′-ClPh-tpy	TBAPF_6_	Fc/Fc^+^		−1.566	0.088	−1.61	tw
5	4′-Cl-tpy	TBAPF_6_	Fc/Fc^+^		−1.498	0.087	−1.541	tw
7	4′-pyrr-tpy	TBAPF_6_	Fc/Fc^+^		−1.840	0.080	−1.880	tw

^a,b^ See footnote of Table 1.

**Table 3:** Electrochemical data (potential in V vs. Fc/Fc^+^) measured in CH_3_CN of the 2nd reduction of bis(terpyridine)Os(II) compounds from this work (tw) at ν = 0.100 Vs^−1^ and published works.

No	Ligand	Electrolyte ^a^	Ref Electrode ^b^	2nd Reduction				Ref
				*E*_1/2_ (Reported)	*E*_1/2_ (Fc/Fc^+^)	∆*E*	*E*_pc_	tw
1	tpy	TBAPF_6_	Fc/Fc^+^		−1.895	0.101	−1.945	tw
	tpy	TEABF_4_	SCE	−1.57	−1.986			[36]
	tpy	TEABF_4_	SCE	−1.52	−1.936			[37]
2	4′-4MePh-tpy	TBAPF_6_	Fc/Fc^+^		−1.881	0.065	−1.913	tw
	4′-4MePh-tpy	TEABF_4_	SCE	−1.54	−1.956			[36]
	4′-4MePh-tpy	TEABF_4_	SCE	−1.54	−1.956			[37]
4	4′-ClPh-tpy	TBAPF_6_	Fc/Fc^+^		−1.826	0.104	−1.878	tw
5	4′-Cl-tpy	TBAPF_6_	Fc/Fc^+^		−1.758	0.085	−1.800	tw
7	4′-pyrr-tpy	TBAPF_6_	Fc/Fc^+^		−2.042	0.093	−2.088	tw

^a,b^ See footnote of Table 1.

**Table 4:** Selected experimental solid-state X-ray and B3LYP/6-311G(d,p)/def2tzvpp solvent (CH_3_CN)-phase-calculated Os-N bond lengths (BL1–BL6 in Å) and angles (ANG1–ANG3 in °) of the indicated bis(terpyridine)osmium(II) compounds. CSD = Cambridge Structural Database.

Complex	ANG1	ANG2	ANG3				Complex
	DFT solvent-phase-computed values						number
[Os(tpy)_2_]^2+^	78.29	92.36	78.29	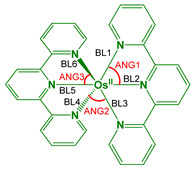			**1**
[Os(4′-4MePh-tpy)_2_]^2+^	78.17	92.41	78.16				**2**
[Os(4,4′,4″-tBu-tpy)_2_]^2+^	78.06	92.38	78.06				**3**
[Os(4′-ClPh-tpy)_2_]^2+^	78.20	92.40	78.20				**4**
[Os(4′-Cl-tpy)_2_]^2+^	78.23	92.38	78.23				**5**
[Os(4′-OMe-tpy)_2_]^2+^	78.24	92.76	78.24				**6**
[Os(4′-pyrr-tpy)_2_]^2+^	78.00	92.53	78.00				**7**
	Experimental values obtained from CSD						CSD ref
[Os(tpy)_2_](BF_4_)_2_	78.56	91.32	78.61				GOLVUA
[Os(tpy)_2_](ClO_4_)_2_	79.32	93.65	79.51				GOGDOV
[Os(tpy)_2_](NO_3_)_2_	78.46	86.83	78.46				KIGWII
	DFT solvent-phase-computed values						Complex
	BL1	BL2	BL3	BL4	BL5	BL6	number
[Os(tpy)_2_]^2+^	2.102	2.009	2.102	2.102	2.009	2.102	**1**
[Os(4′-4MePh-tpy)_2_]^2+^	2.103	2.007	2.103	2.103	2.007	2.103	**2**
[Os(4,4′,4″-tBu-tpy)_2_]^2+^	2.102	2.007	2.101	2.102	2.008	2.101	**3**
[Os(4′-ClPh-tpy)_2_]^2+^	2.103	2.006	2.103	2.103	2.006	2.103	**4**
[Os(4′-Cl-tpy)_2_]^2+^	2.104	2.008	2.104	2.104	2.008	2.104	**5**
[Os(4′-OMe-tpy)_2_]^2+^	2.102	2.013	2.102	2.102	2.013	2.102	**6**
[Os(4′-pyrr-tpy)_2_]^2+^	2.101	2.017	2.101	2.101	2.017	2.101	**7**
	Experimental values obtained from CSD ^a^						CSD ref
[Os(tpy)_2_](BF_4_)_2_	2.082	1.993	2.078	2.076	2.005	2.083	GOLVUA
[Os(tpy)_2_](ClO_4_)_2_	2.07	1.98	2.053	2.062	1.964	2.05	GOGDOV
[Os(tpy)_2_](NO_3_)_2_	2.069	1.988	2.05	2.05	1.988	2.069	KIGWII

^a^. AD (Average deviation) of DFT calculated from experimental for (1): Os-N_terminal_, (BL1, BL3, BL4, BL6) = 0.036 Å, Os-N_central_, (BL2, BL5) = 0.023 Å. ^a^. MAD (Mean Average Deviation) of DFT calculated from experimental for (1): Os-N_terminal_, (BL1, BL3, BL4, BL6) = 0.011 Å, Os-N_central_, (BL2, BL5) = 0.010 Å.

**Table 5:** B3LYP/6-311G(d,p)/def2tzvpp solvent (CH_3_CN)-phase-calculated energies (eV) for the oxidized and reduced bis(terpyridine)osmium(II); [Os(tpy)_2_]^m^, m = 0–3.

m	S	ΔE (eV)	m	S	ΔE (eV)
3	1/2	0.000	2	0	0.000
	3/2	2.103		1	1.841
	5/2	5.066		2	3.934
1	1/2	0.000	0	0	0.010
	3/2	1.543		1	0.000

**Table 6:** B3LYP/6-311G(d,p)/def2tzvpp solvent (CH_3_CN) phase-computed Os-N bond lengths (Å) of ground states of [Os(tpy)_2_]^m^*,* m = 0–3. Os-N_terminal_ (BL1, BL3, BL4, BL6) and Os-N_central_ (BL2, BL5) bond lengths and angles (ANG1-ANG3) are indicated in Table 4.

m	S	ANG1	ANG2	ANG3	BL1	BL2	BL3	BL4	BL5	BL6
0	1	77.77	91.53	77.77	2.092	2.013	2.095	2.092	2.013	2.095
1	1/2	78.55	92.42	78.87	2.093	2.031	2.105	2.092	1.985	2.093
2	0	78.29	92.36	78.29	2.102	2.009	2.102	2.102	2.009	2.102
3	1/2	77.61	95.89	78.4	2.101	2.038	2.101	2.114	2.016	2.107

**Table 7:** Mulliken spin density (e^−^) on Os and the two tpy ligands of the ground states of [Os(tpy)_2_]^m^*,* m = 0, 1, and 3.

m	S	Mulliken Spin Density		
		Os	Ligand 1	Ligand 2
0	1	0.2212	0.8894	0.8894
1	1/2	0.0890	0.8571	0.0539
3	1/2	0.8559	0.0567	0.0874

**Table 8:** Hammett substituent constants (from [51] for (**1**)–(**7**)), *E*_1/2,ox_ (vs. Fc/Fc^+^) and DFT solvent phase (CH_3_CN)-computed energies (eV) for (**1**)–(**15**). Ligand abbreviations given in Scheme 1 and Scheme 2. *E*_1/2,ox_ of (**8**)–(**15**) from [28].

No	Ligand	*E*_1/2,ox_	*E*_HOMO_	*E*_LUMO_	*χ*	*ω*	*E*(III)-*E*(II)	*E*(II)-*E*(I)	*G*(III)-*G*(II)	*G*(II)-*G*(I)	Σσ*_p_*
**1**	tpy	0.546	−6.076	−2.852	4.464	3.090	5.636	8.741	5.677	8.993	0.00
**2**	4′-4MePh-tpy	0.495	−6.031	−2.856	4.443	3.109	5.550	8.659	5.651	8.930	−0.03
**3**	4,4′,4′′-tBu-tpy	0.394	−5.844	−2.644	4.244	2.814	5.399	-	5.493	-	−0.60
**4**	4′-ClPh-tpy	0.545	−6.104	−2.916	4.510	3.190	5.616	8.785	5.634	9.057	0.12
**5**	4′-Cl-tpy	-	−6.192	−2.978	4.585	3.271	5.734	8.991	5.786	9.201	0.23
**6**	4′-OMe-tpy	0.398	−5.894	−2.748	4.321	2.967	5.425	8.453	5.409	8.638	−0.27
**7**	4′-pyrr-tpy	0.051	−5.435	−2.551	3.993	2.763	5.018	7.831	5.015	8.026	−0.83
**8**	4Mephen	0.356	−5.823	−2.737	4.280	2.969	5.382		5.413		
**9**	5Clphen	0.535	−6.076	−3.005	4.540	3.357	5.634		5.662		
**10**	bpy	0.452	−5.912	−2.883	4.397	3.192	5.481		5.498		
**11**	dMebpy	0.286	−5.707	−2.746	4.226	3.017	5.269		5.345		
**12**	dOMebpy	0.074	−5.464	−2.592	4.028	2.824	5.032		5.086		
**13**	dtBubpy	0.298	−5.712	−2.725	4.218	2.978	5.272		5.292		
**14**	phen	0.444	−5.925	−2.842	4.384	3.116	5.496		5.535		
**15**	TetraMephen	0.213	−5.622	−2.503	4.062	2.645	5.189		5.120		

**Table 9:** DFT solvent (CH_3_CN)-calculated NBO charges Q (e^−^) and MESP charge Q (e^−^) and MESP V (au) for complexes (**1**)–(**15**). Ligand abbreviations are given in Scheme 1 and Scheme 2.

No	Ligand	*Q*_NBO(Os)_	*Q*_NBO(Os+N)_	*Q*_MESP(Os)_	*Q*_MESP(Os+N)_	*V*_MESP(Os)_	*V*_MESP(Os+N)_
**1**	tpy	0.6589	−1.8341	−0.4266	0.0271	−12.0812	−12.0812
**2**	4′-4MePh-tpy	0.6606	−1.8424	−0.2753	−0.0471	−12.0886	−12.0886
**3**	4,4′,4′′-tBu-tpy	0.6515	−1.8597	−0.2267	−0.2140	−12.1047	−12.1047
**4**	4′-ClPh-tpy	0.6631	−1.8347	−0.3139	0.0346	−12.0776	−12.0776
**5**	4′-Cl-tpy	0.6626	−1.8312	−0.3236	−0.0148	−12.0687	−12.0687
**6**	4′-OMe-tpy	0.6455	−1.8843	−0.0979	−0.3047	−12.0944	−12.0944
**7**	4′-pyrr-tpy	0.6361	−1.9342	−0.2220	−0.1760	−12.1183	−12.1183
**8**	4Mephen	0.5639	−1.9312	0.0702	−0.4930	−12.1086	−12.1086
**9**	5Clphen	0.5717	−1.9003	−0.1047	−0.1895	−12.0818	−12.0818
**10**	bpy	0.5712	−1.9548	−0.3498	−0.0247	−12.0951	−12.0951
**11**	dMebpy	0.5656	−1.9917	−0.1528	−0.4280	−12.1136	−12.1136
**12**	dOMebpy	0.5464	−2.1023	0.0394	−0.7312	−12.1296	−12.1296
**13**	dtBubpy	0.5653	−1.9743	−0.1495	−0.3061	−12.1176	−12.1176
**14**	phen	0.5679	−1.9104	−0.0236	−0.3180	−12.0993	−12.0993
**15**	TetraMephen	0.5593	−1.9194	0.3969	−0.6642	−12.1286	−12.1286

## Data Availability

Data are within the manuscript and Supplementary Materials.

## Supplementary Materials

The following supporting information can be downloaded at: https://www.mdpi.com/article/10.3390/molecules29215078/s1, Figures S1–S7: ^1^H NMR; Figures S8–S13 ^13^C NMR, Figures S14–S20 UV/vis spectra, Figure S21 FTIR spectra, Figures S22–S28 PXRD spectra. Optimized coordinates of the DFT calculations.
