# Supplementary material for: Synthesis, Electrochemistry and Density Functional Theory of Osmium(II) Containing Different 2,2′:6′,2″-Terpyridines
Source: Molecules. 2024 Oct 27;29(21):5078. doi: 10.3390/molecules29215078 (PMC11547826; doi:10.3390/molecules29215078)
Supplement: Supplementary file 1 [file molecules-29-05078-s001.zip › molecules-3262424-supplementary.pdf]

# Synthesis, Electrochemistry and Density Functional Theory of Osmium(II) Containing Different 2,2′:6′,2″-Terpyridines

## Supplementary Materials

## Contents


1 $^{1}$H NMR SPECTRA ....... 2

2 $^{13}$C NMR SPECTRA ....... 5

3 UV/VISIBLE SPECTRA ....... 9

4 FTIR SPECTRA ....... 12

5 PXRD ....... 13

6 OPTIMIZED COORDINATES ....... 17

6.1 [Os (2,2′:6′,2″-terpyridine)$_2$]$^{2+}$ ....... 17

6.2 [Os (4'-(4-methylphenyl)-2,2′:6′,2″-terpyridine)$_2$]$^{2+}$ ....... 18

6.3 [Os (4,4’,4"-tri-tert-Butyl-2,2′:6′,2″-terpyridine)$_2$]$^{2+}$ ....... 20

6.4 [Os (4'-(4-chlorophenyl)-2,2′:6′,2″-terpyridine)$_2$]$^{2+}$ ....... 22

6.5 [Os (4’-chloro-2,2′:6′,2″-terpyridine)$_2$]$^{2+}$ ....... 24

6.6 [Os (4’-methoxy)-2,2′:6′,2″-terpyridine)$_2$]$^{2+}$ ....... 25

6.7 [Os 4'-(N-Pyrrolidinyl)-2,2′:6′,2″-terpyridine)$_2$]$^{2+}$ ....... 26

7 REFERENCES ....... 28

# 1 $^{1}$H NMR spectra

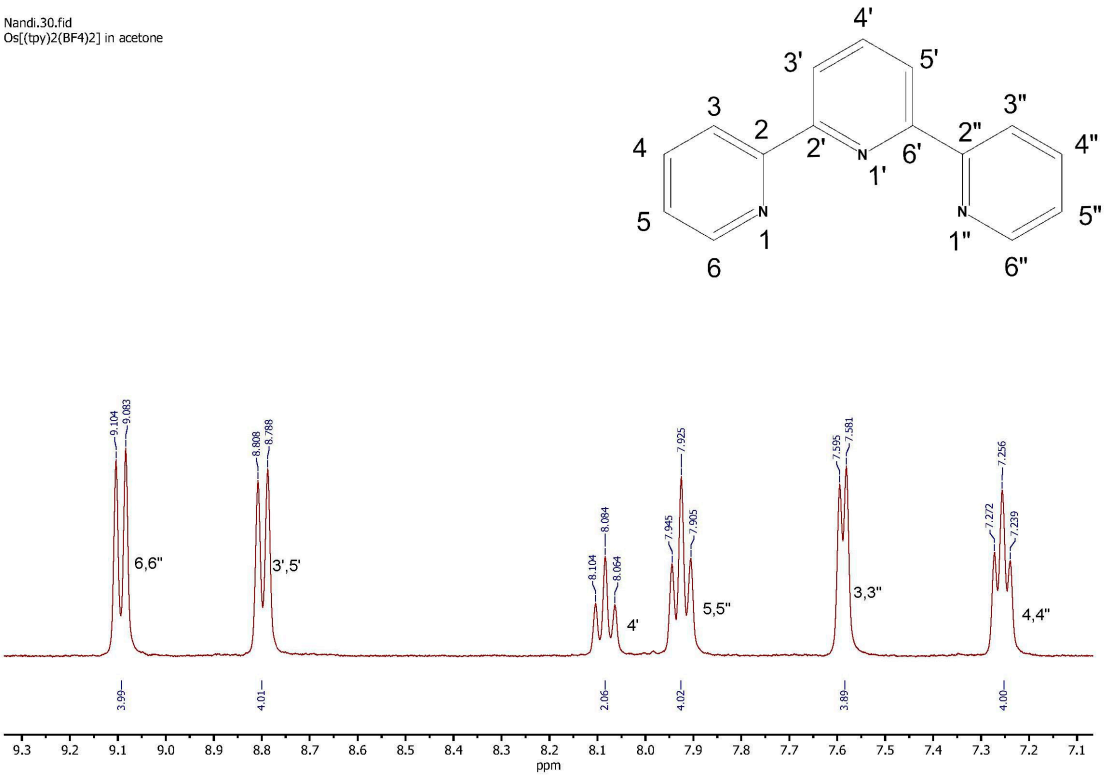


**Figure S 1.** $^{1}$**H NMR of [Os (2,2′:6′,2″-terpyridine)$_2$](BF$_4$)$_2$ (1).**

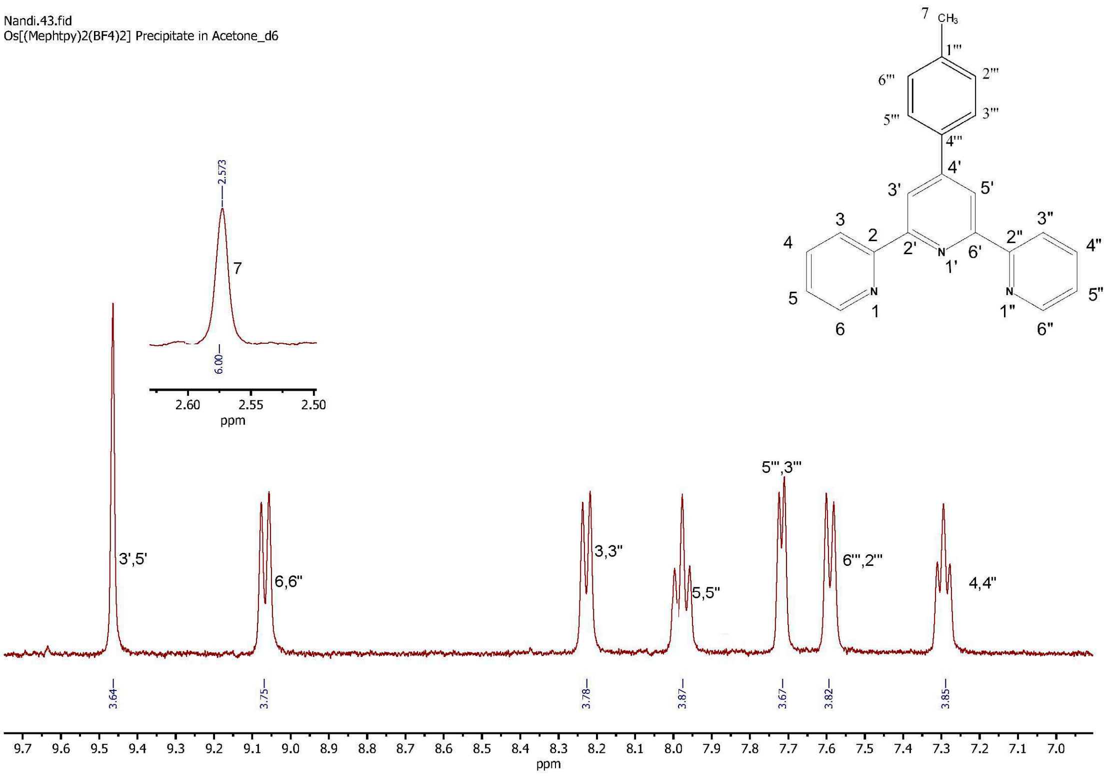


**Figure S 2.** $^{1}$**H NMR of [Os (4'-(4-methylphenyl)-2,2′:6′,2″-terpyridine)$_2$](BF$_4$)$_2$ (2).**

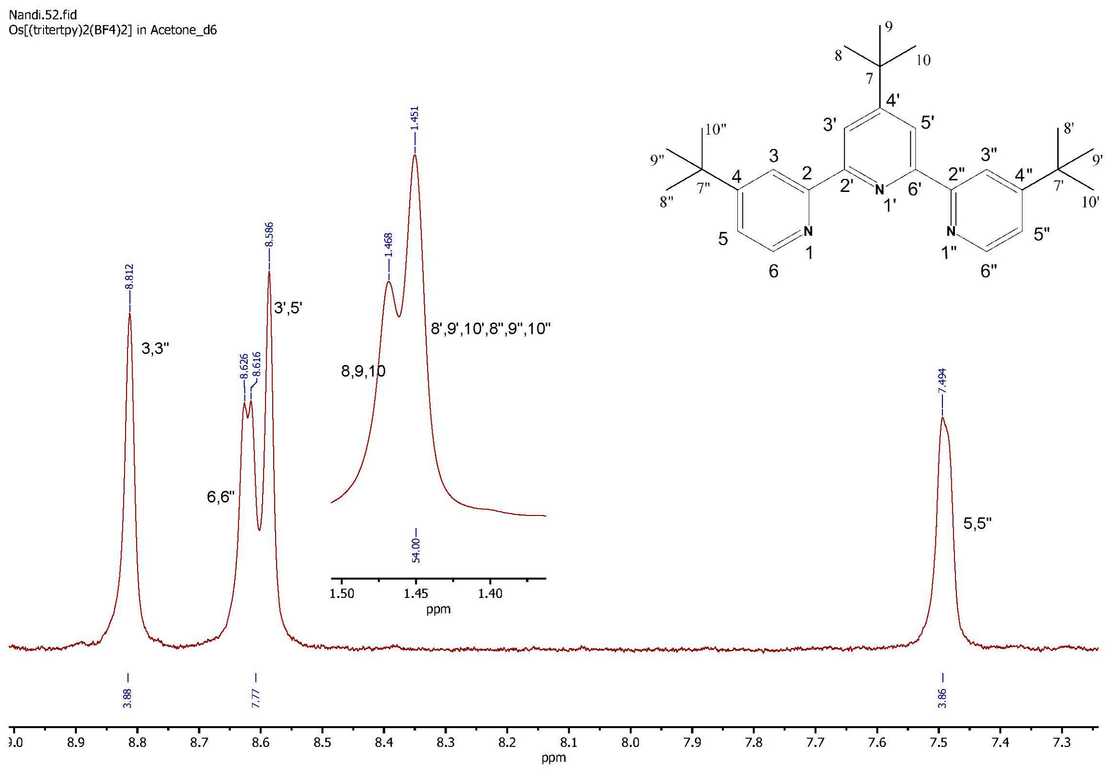


**Figure S 3. $^{1}$H NMR of [Os (4,4',4"-tri-tert-Butyl-2,2′:6′,2″-terpyridine)$_2$]($BF_4$)$_2$ (3).**

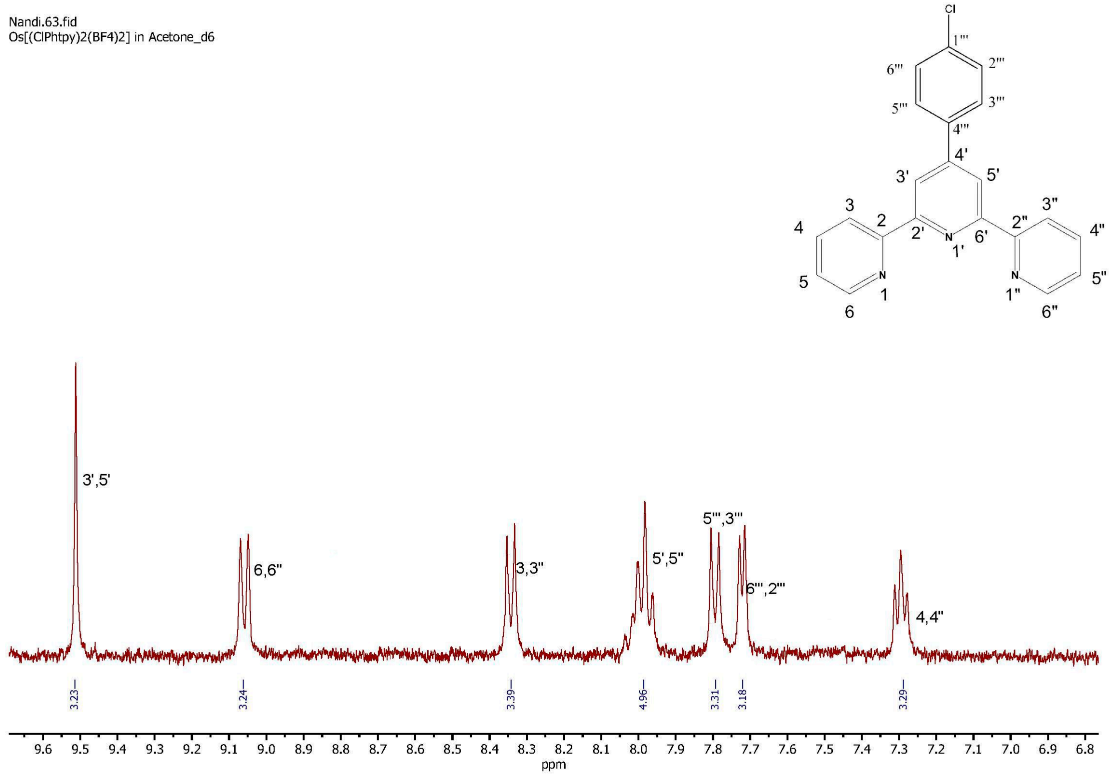


**Figure S 4. $^{1}$H NMR of [Os (4'-(4-chlorophenyl)-2,2′:6′,2″-terpyridine)$_2$]($BF_4$)$_2$ (4).**

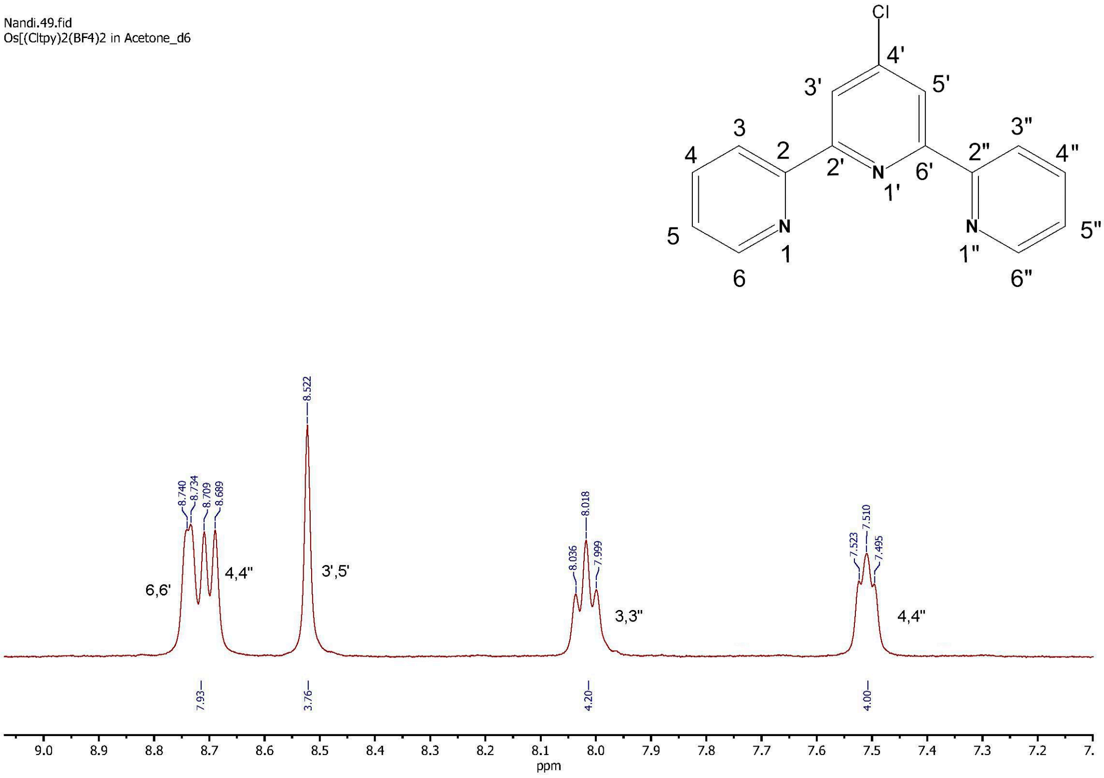


**Figure S 5.** **$^{1}$H NMR of [Os (4'-chloro-2,2′:6′,2″-terpyridine)$_2$](BF$_4$)$_2$ (5).**

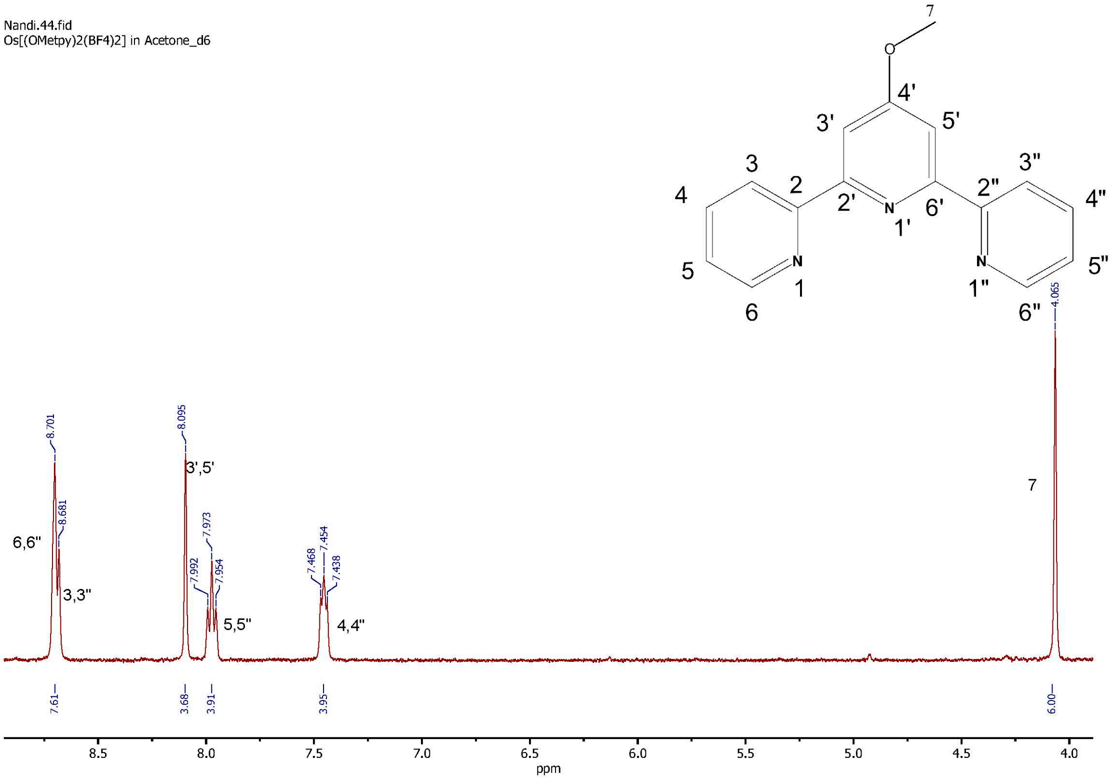


**Figure S 6.** **$^{1}$H NMR of [Os (4'-methoxy)-2,2′:6′,2″-terpyridine)$_2$](BF$_4$)$_2$ (6).**

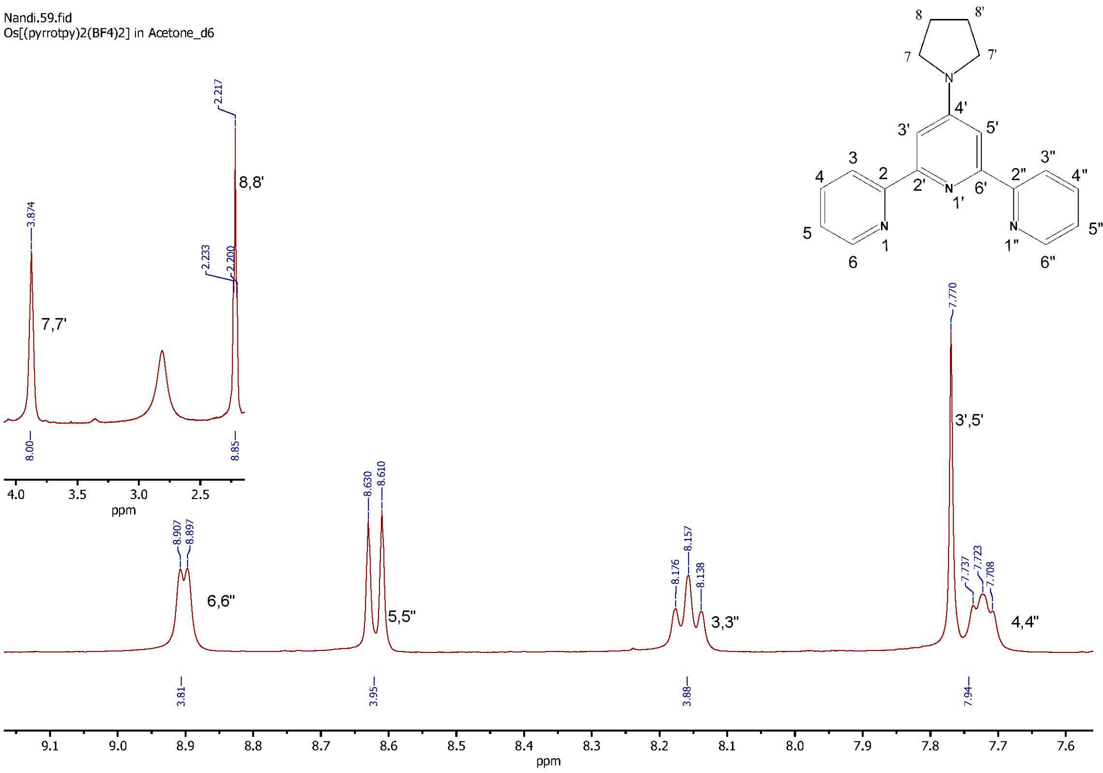


**Figure S 7.** **$^{1}$H NMR of [Os 4'-(N-Pyrrolidinyl)-2,2′:6′,2″-terpyridine)$_2$](BF$_4$)$_2$ (7).**

# 2 $^{13}$C NMR spectra

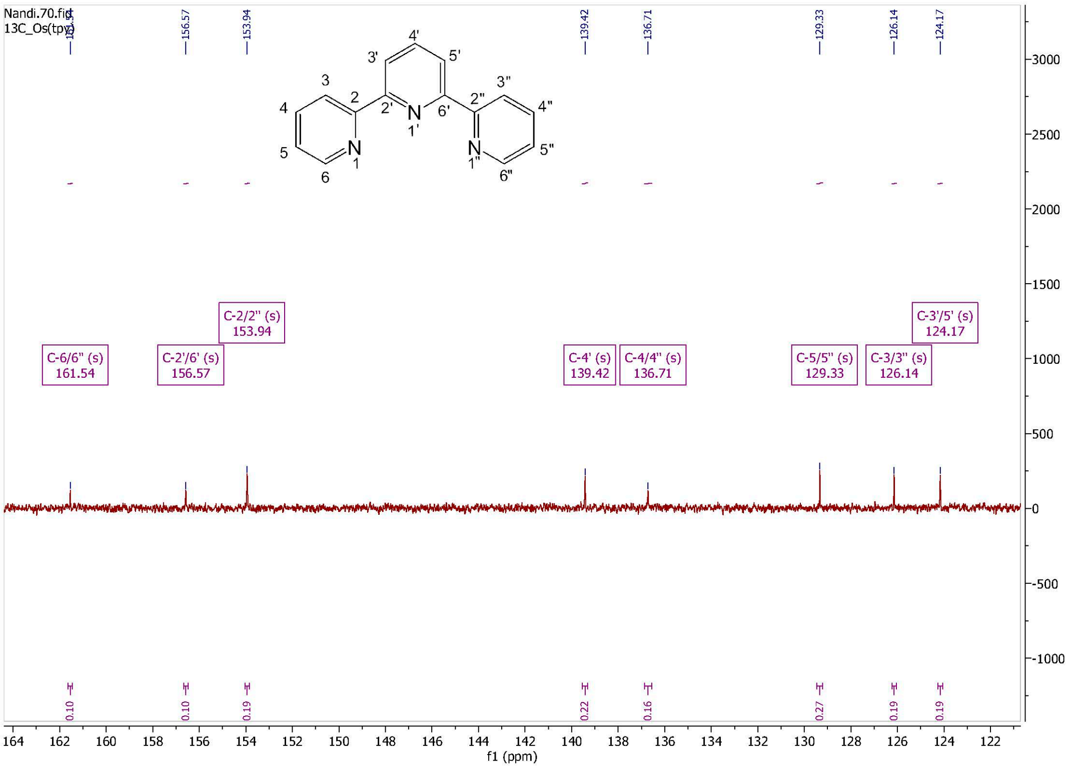


**Figure S 8.** **$^{13}$C NMR of [Os (2,2′:6′,2″-terpyridine)$_2$](BF$_4$)$_2$ (1).**

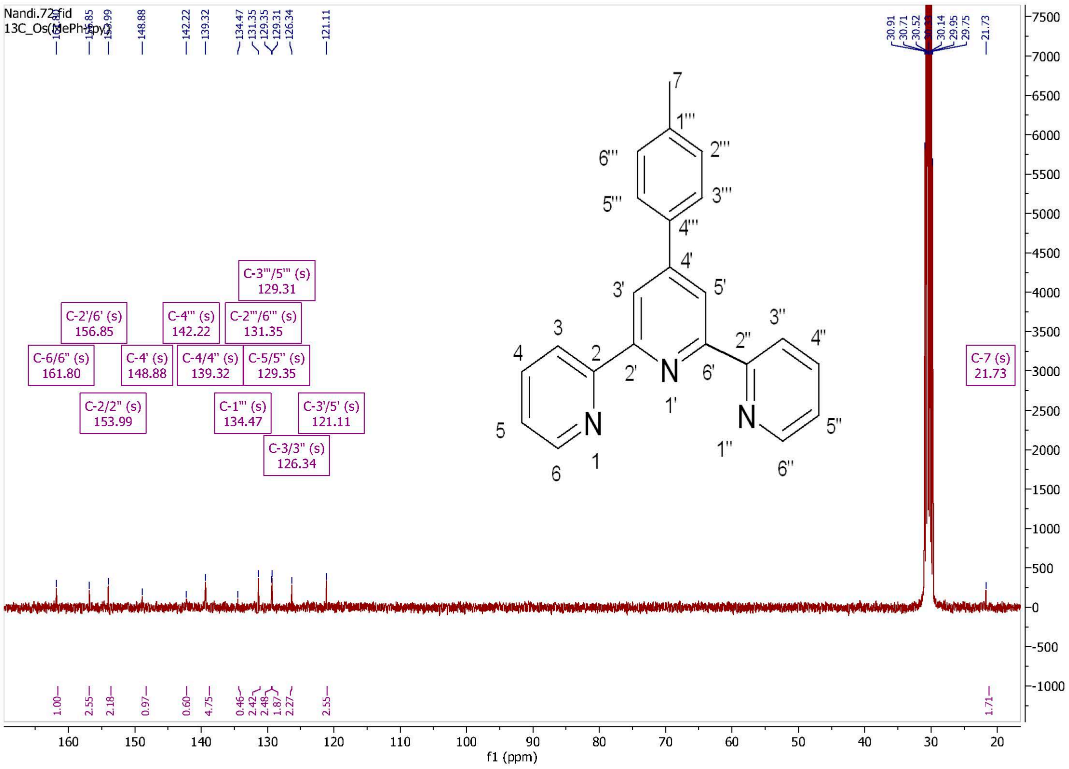

**Figure S 9.** $^{13}$**C NMR of [Os (4'-(4-methylphenyl)-2,2′:6′,2″-terpyridine)$_2$](BF$_4$)$_2$** (2).

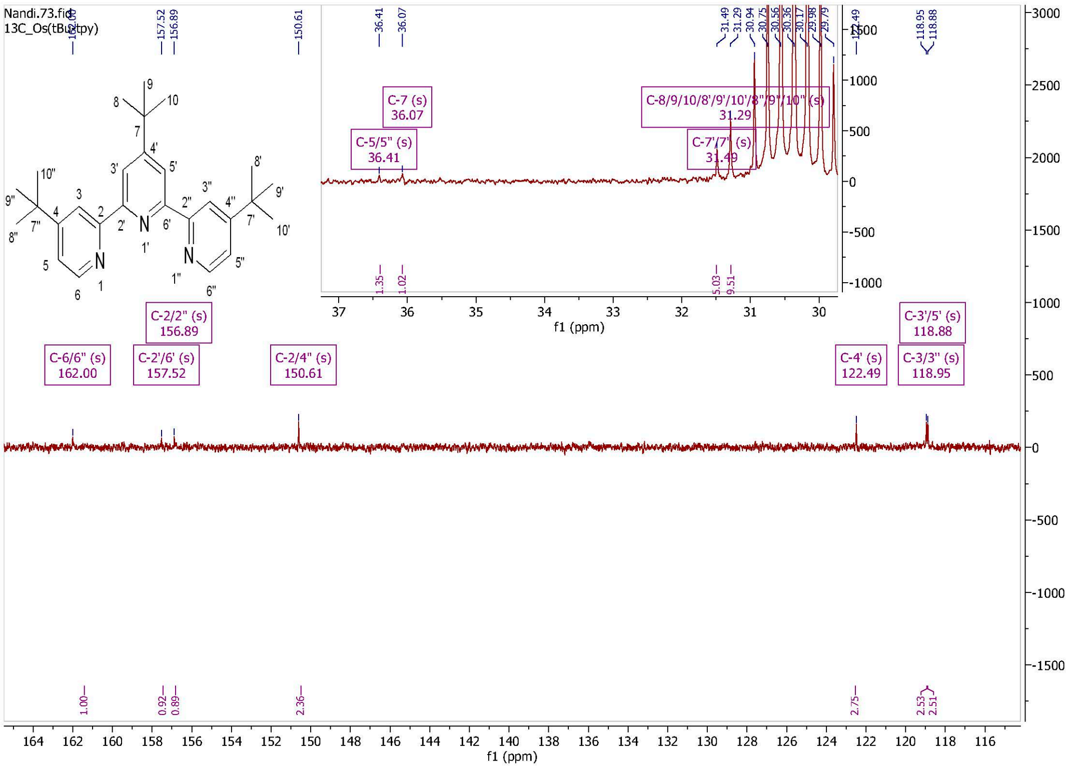

**Figure S 10.** $^{13}$**C NMR of [Os (4,4’,4"-tri-tert-Butyl-2,2′:6′,2″-terpyridine)$_2$](BF$_4$)$_2$** (3).

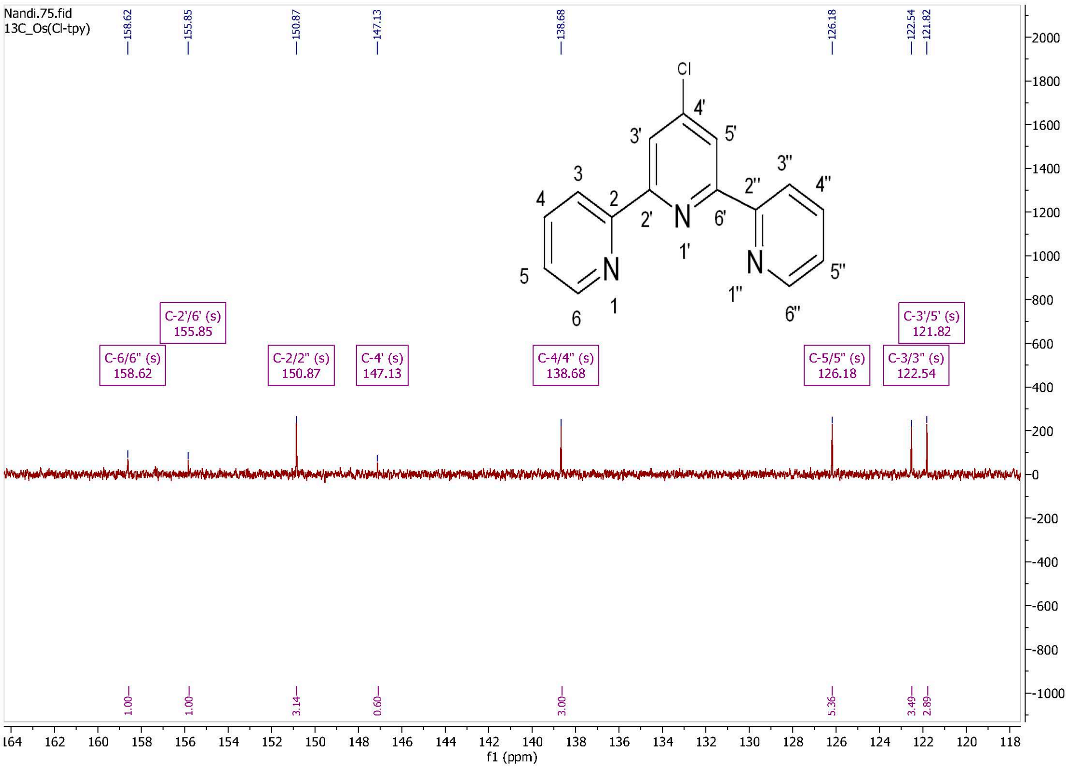


**Figure S 11.** **$^{13}$C NMR of [Os (4’-chloro-2,2′:6′,2″-terpyridine)$_2$](BF$_4$)$_2$ (5).**

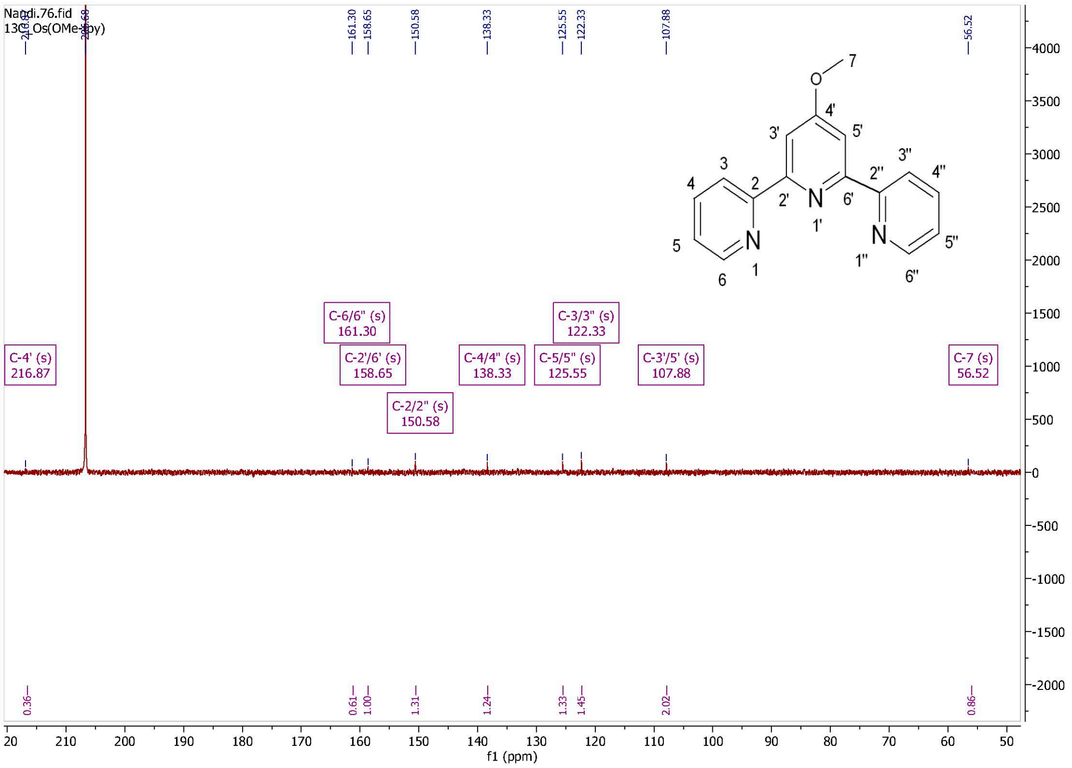


**Figure S 12.** **$^{13}$C NMR of [Os (4’-methoxy)-2,2′:6′,2″-terpyridine)$_2$](BF$_4$)$_2$ (6).**

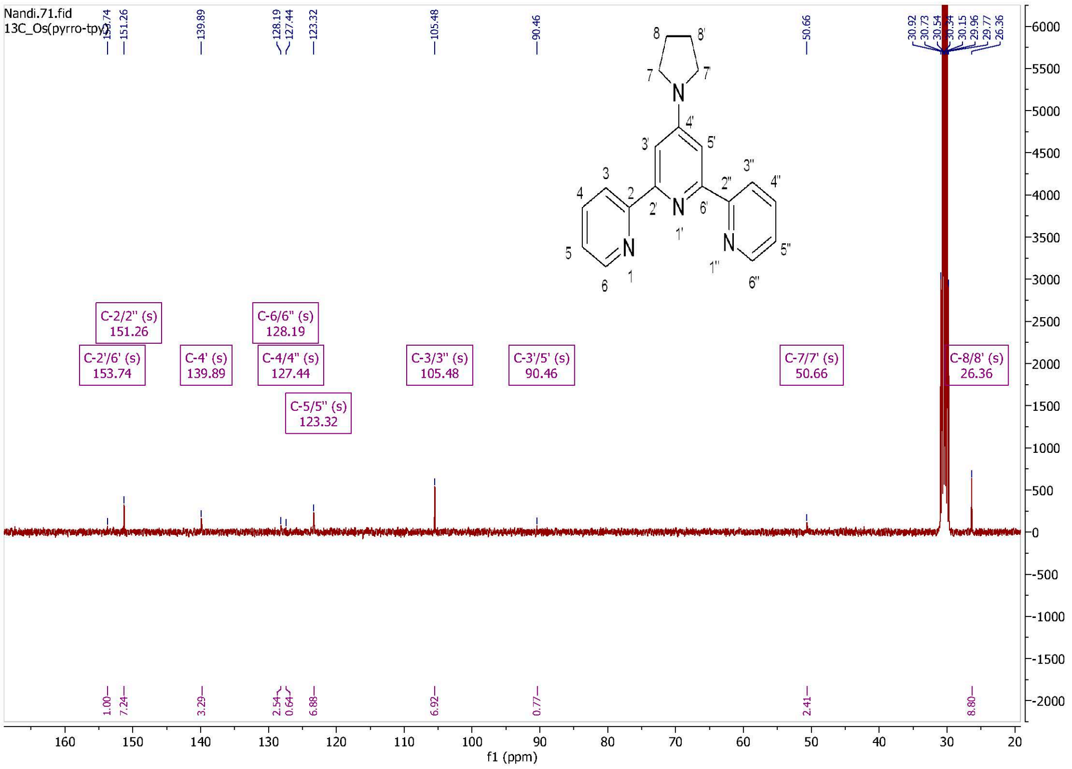


**Figure S 13. $^{13}$C NMR of [Os 4'-(N-Pyrrolidinyl)-2,2′:6′,2″-terpyridine)$_2$](BF$_4$)$_2$ (7).**

## 3 UV/Visible spectra

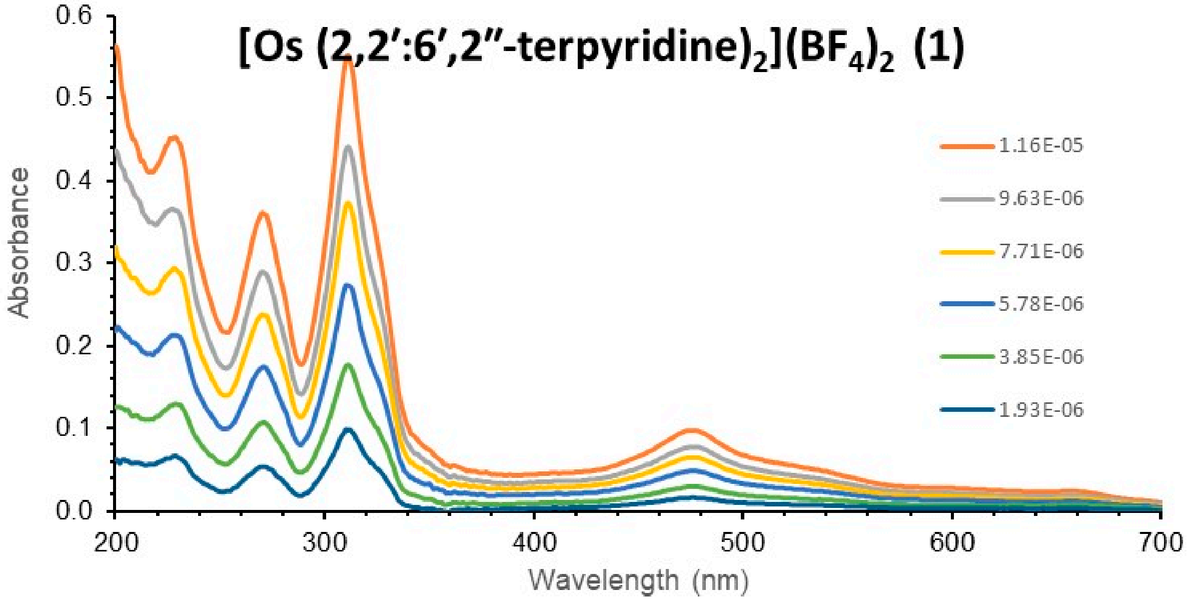


**Figure S 14.** UV/Visible spectra of [Os (2,2′:6′,2″-terpyridine)$_2$](BF$_4$)$_2$ (1). Concentration is indicated in mol dm$^{-3}$.

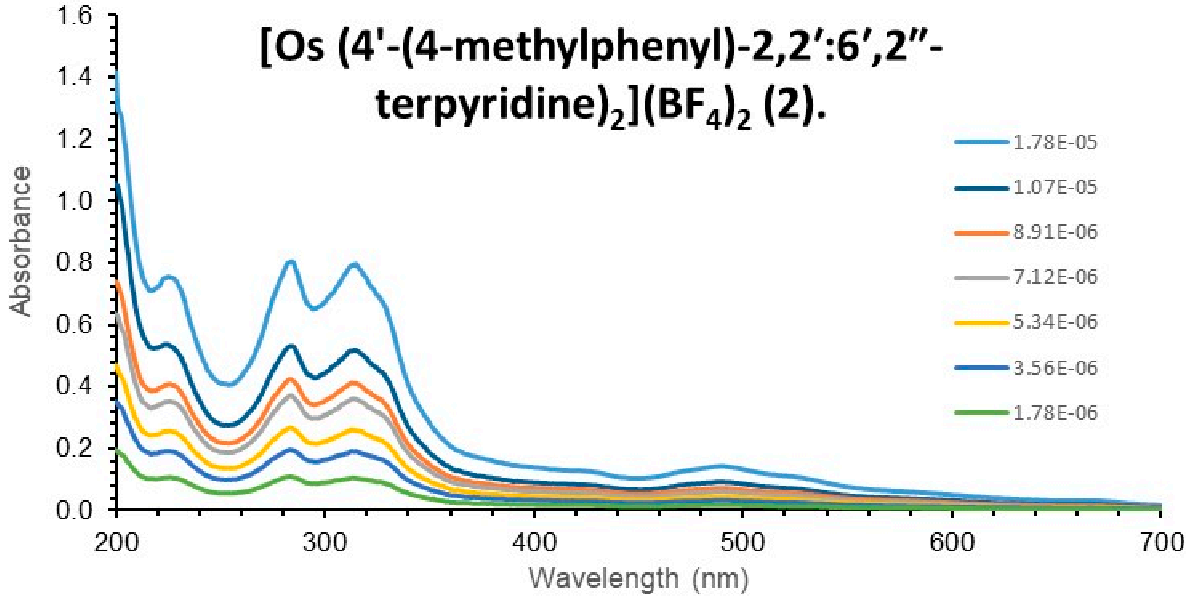


**Figure S 15.** UV/Visible spectra of [Os (4'-(4-methylphenyl)-2,2′:6′,2″-terpyridine)$_2$](BF$_4$)$_2$ (2). Concentration is indicated in mol dm$^{-3}$.

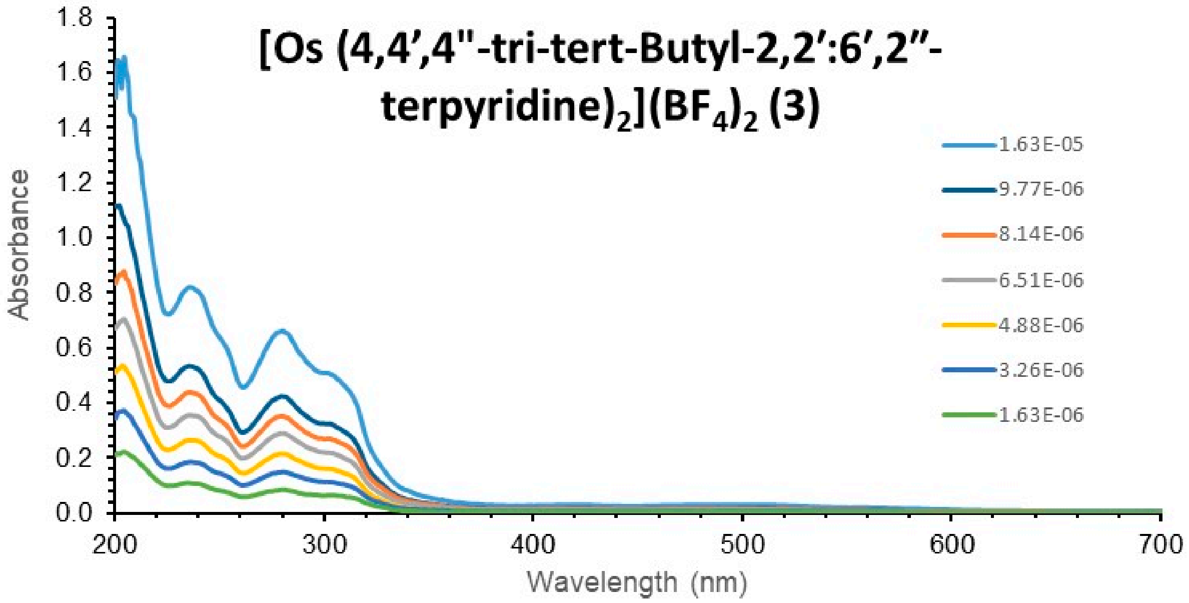


**Figure S 16.** **UV/Visible spectra of [Os (4,4',4"-tri-tert-Butyl-2,2′:6′,2″-terpyridine)$_2$](BF$_4$)$_2$ (3). Concentration is indicated in mol dm$^{-3}$.**

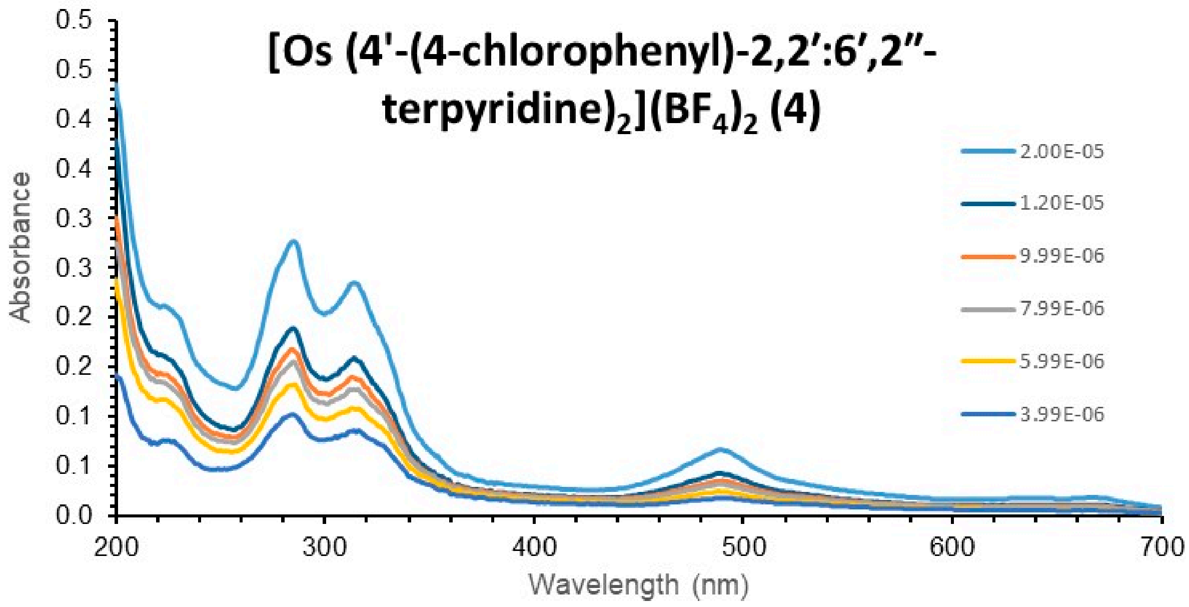


**Figure S 17.** **UV/Visible spectra of [Os (4'-(4-chlorophenyl)-2,2′:6′,2″-terpyridine)$_2$](BF$_4$)$_2$ (4). Concentration is indicated in mol dm$^{-3}$.**

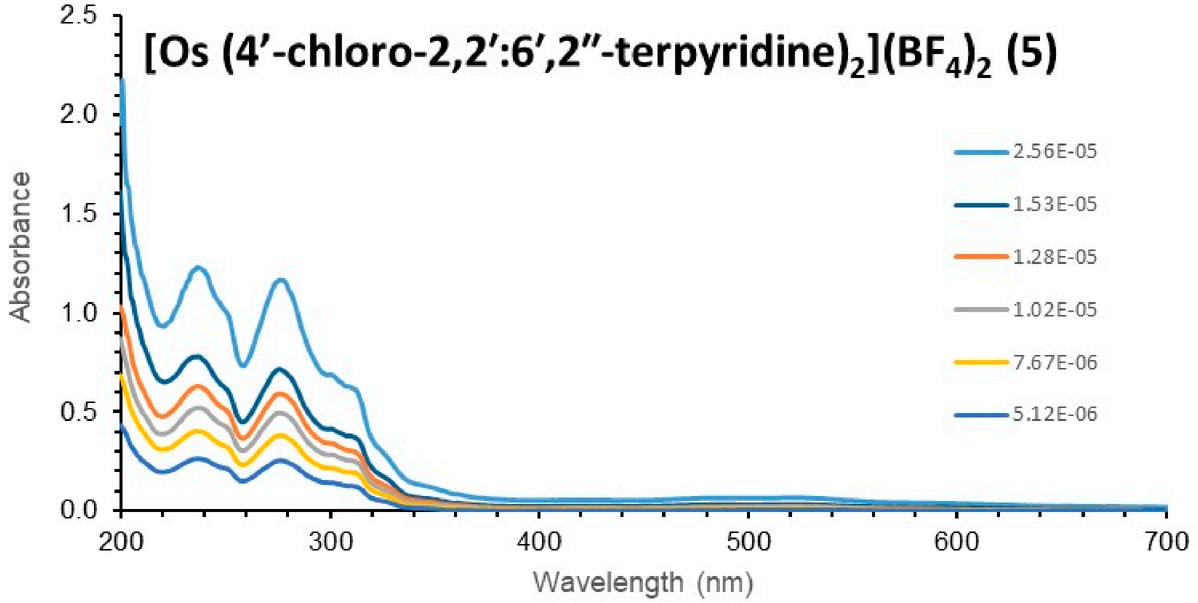


**Figure S 18. UV/Visible spectra of [Os (4'-chloro-2,2′:6′,2″-terpyridine)$_2$](BF$_4$)$_2$ (5). Concentration is indicated in mol dm$^{-3}$.**

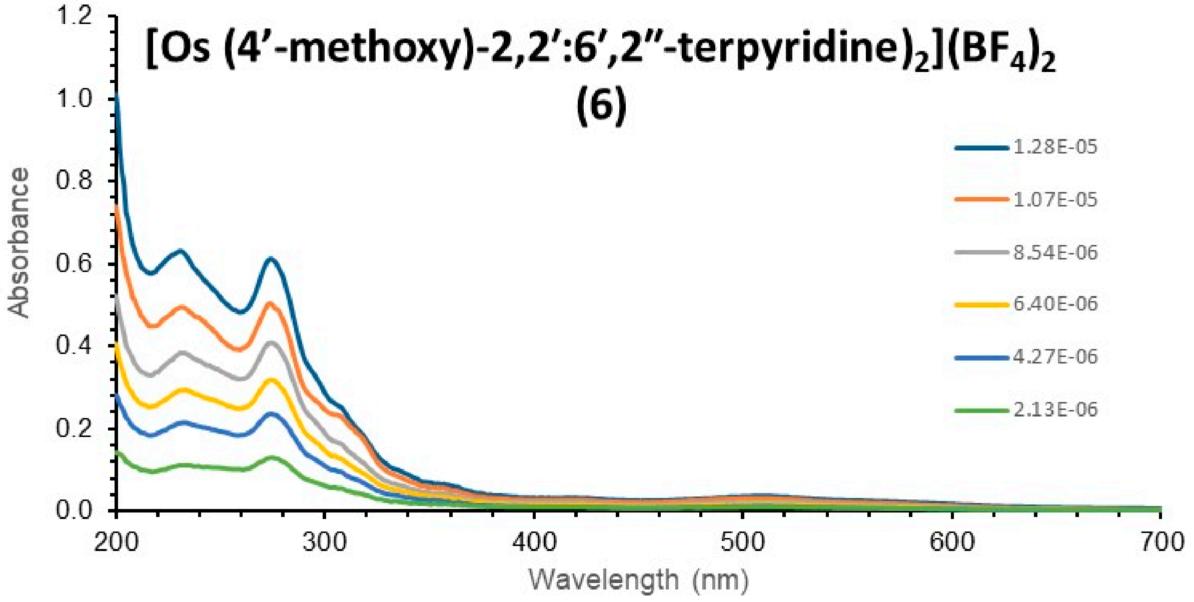


**Figure S 19. UV/Visible spectra of [Os (4'-methoxy)-2,2′:6′,2″-terpyridine)$_2$](BF$_4$)$_2$ (6). Concentration is indicated in mol dm$^{-3}$.**

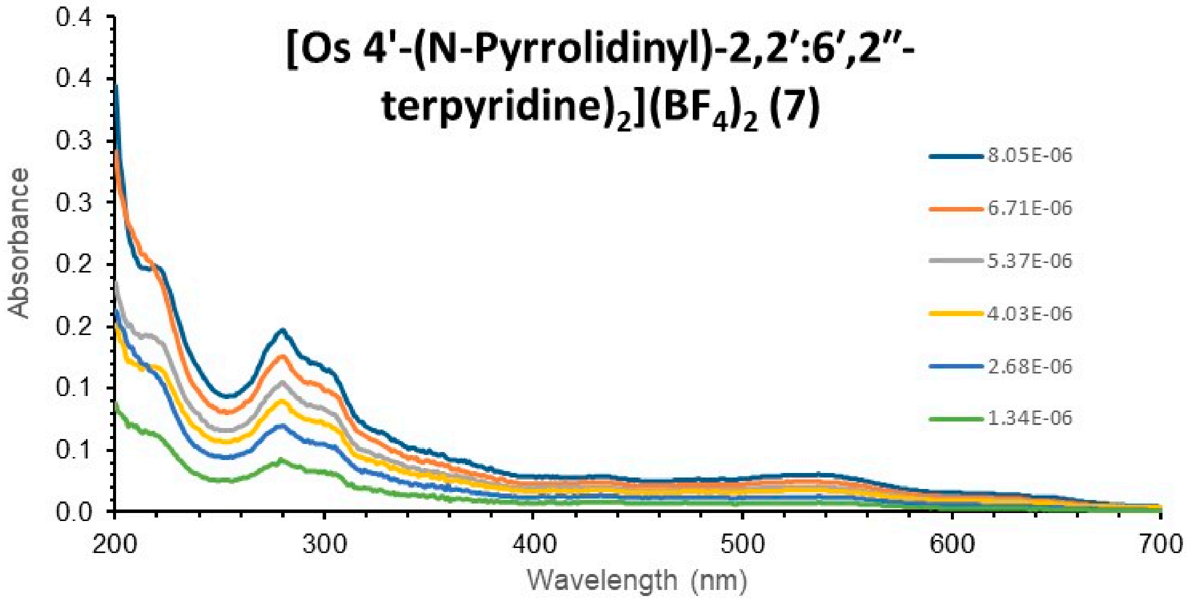


**Figure S 20. UV/Visible spectra of [Os 4'-(N-Pyrrolidinyl)-2,2′:6′,2″-terpyridine)$_2$](BF$_4$)$_2$ (7). Concentration is indicated in mol dm$^{-3}$.**

# 4 FTIR spectra

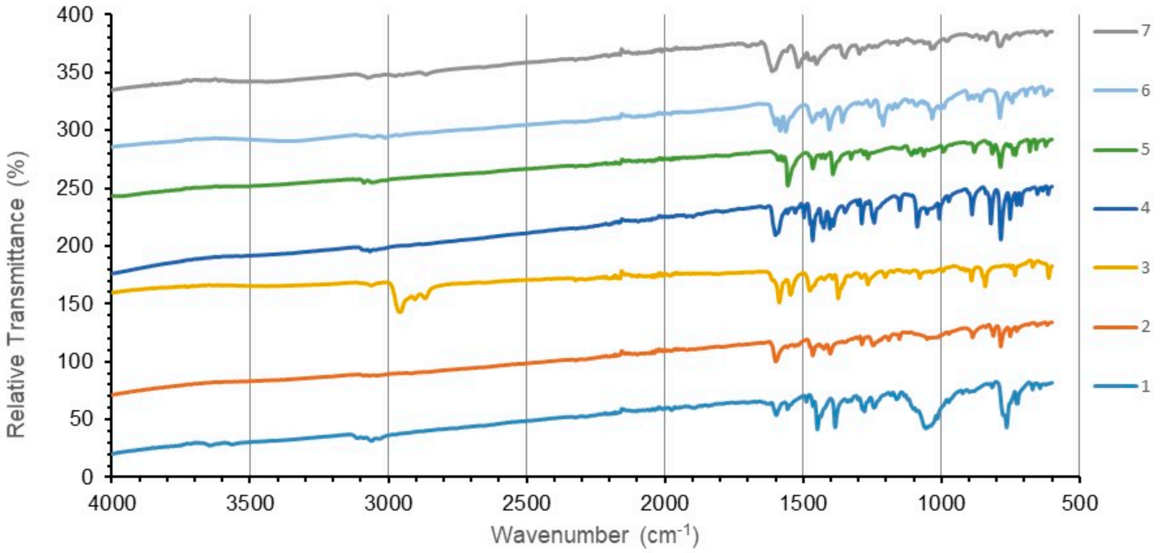


**Figure S 21. Experimental IR spectra of (1) – (7).**

## 5 PXRD

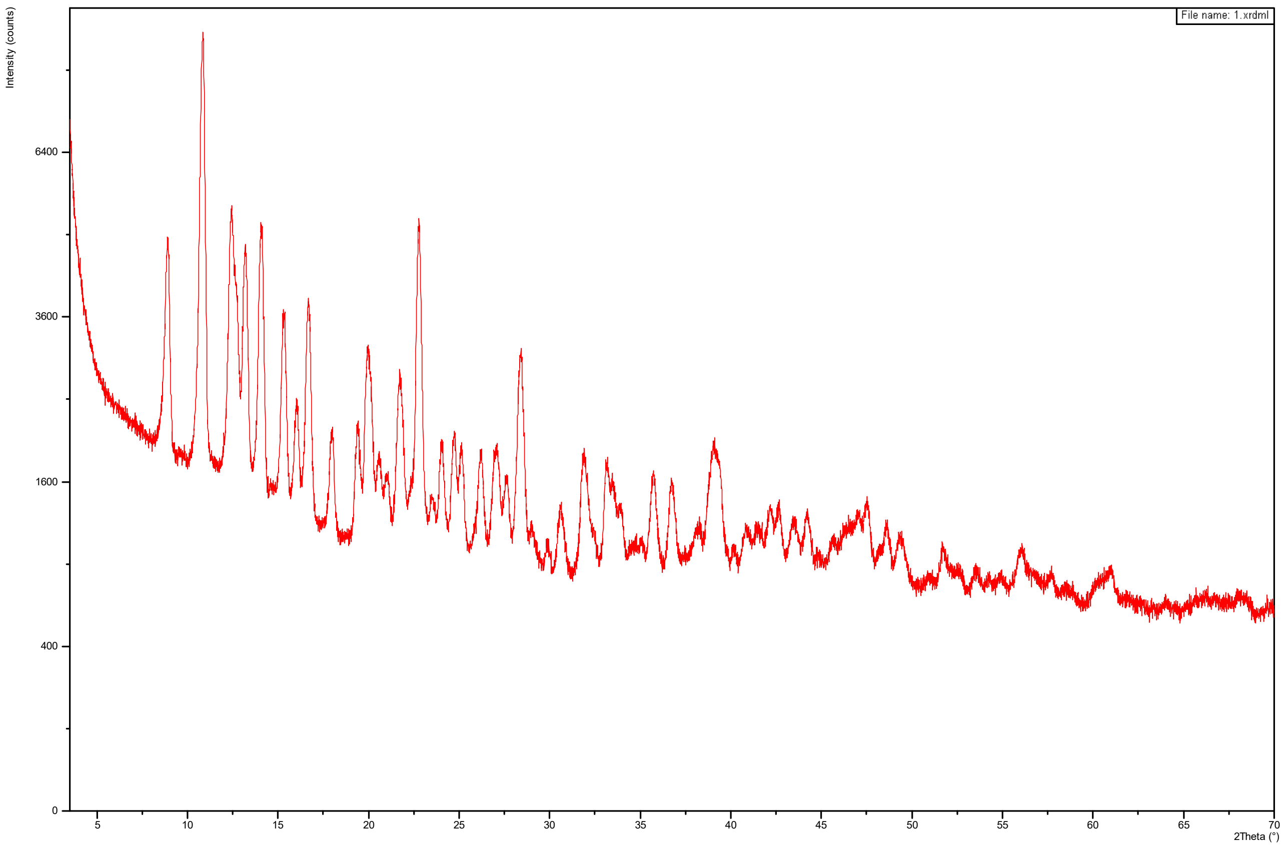


**Figure S 22. XRD of [Os (2,2′:6′,2″-terpyridine)$_2$](BF$_4$)$_2$ (1).**

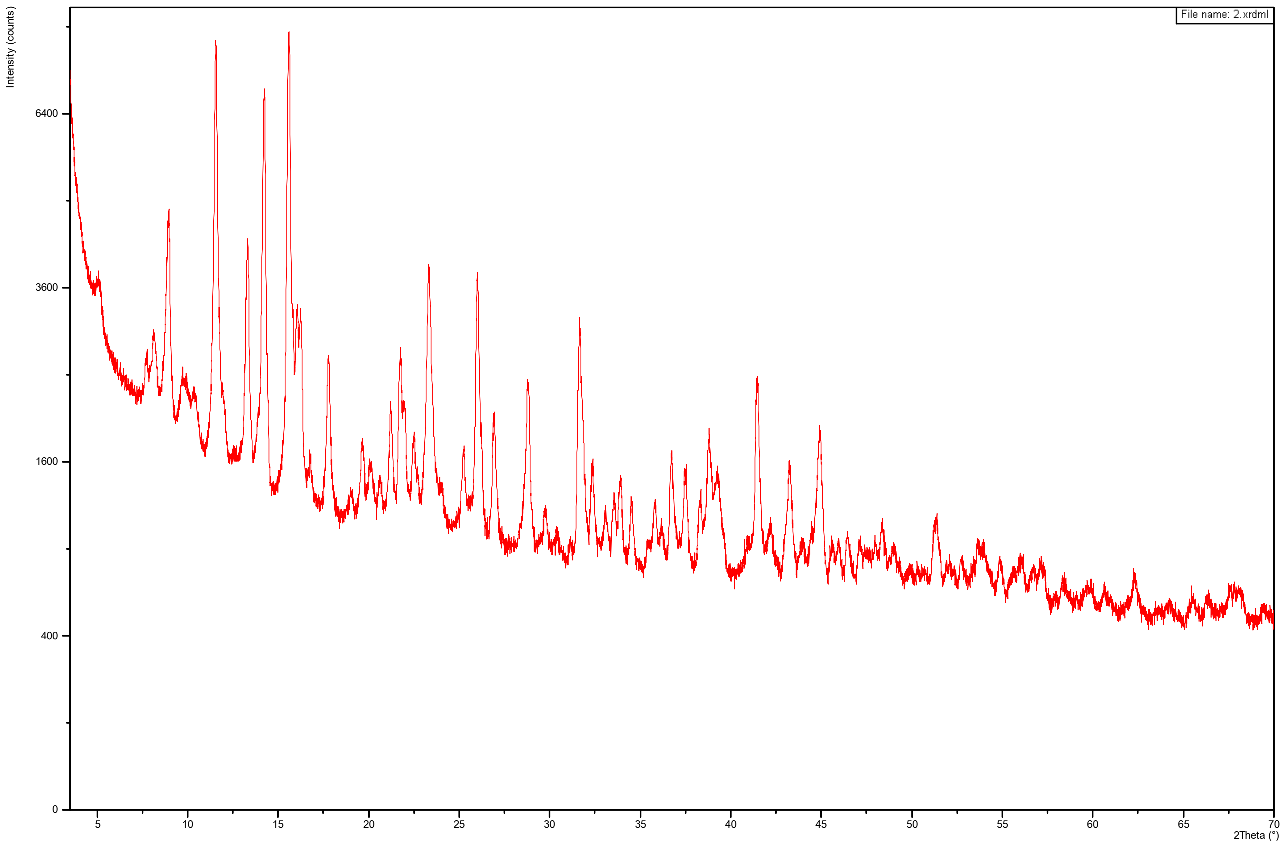


**Figure S 23. XRD of [Os (4'-(4-methylphenyl)-2,2′:6′,2″-terpyridine)$_2$](BF$_4$)$_2$ (2).**

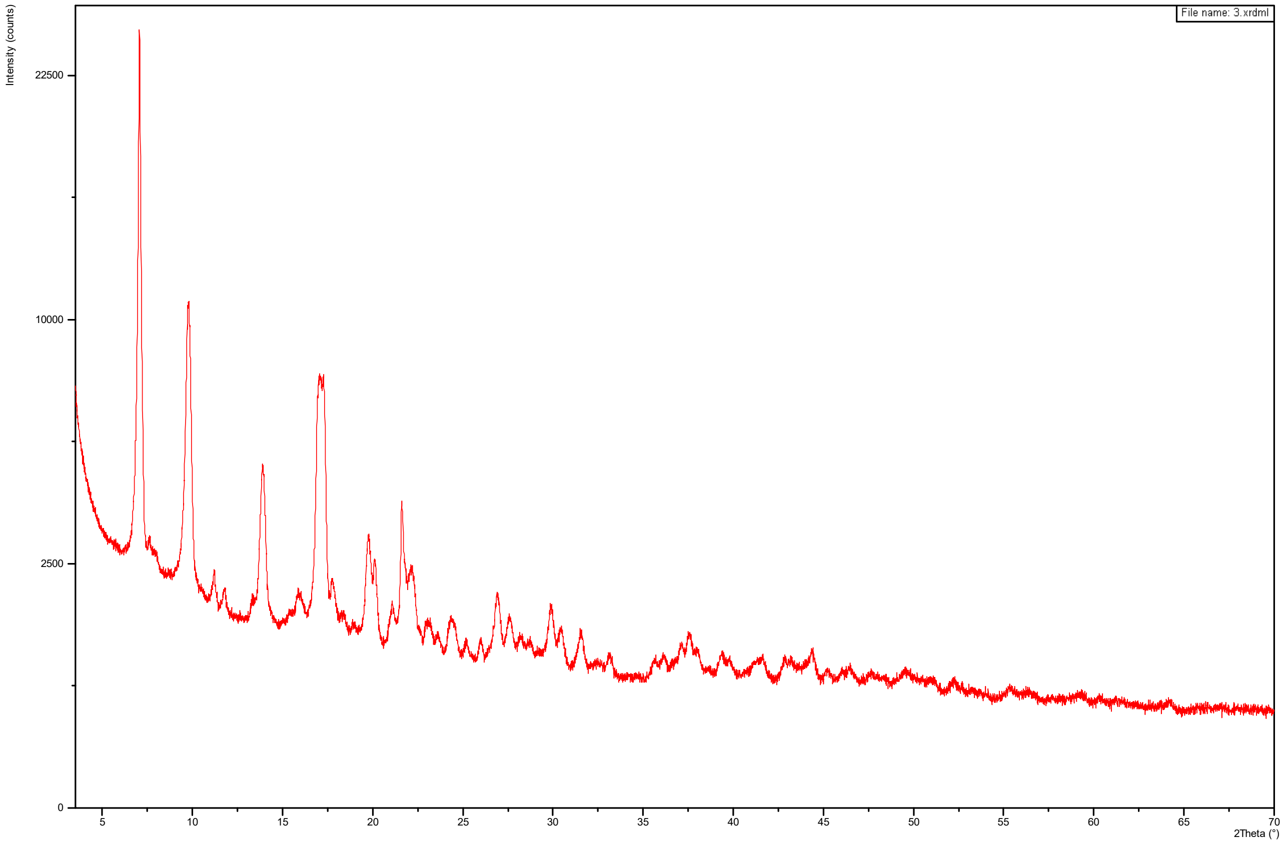


**Figure S 24. XRD of [Os (4,4’,4"-tri-tert-Butyl-2,2′:6′,2″-terpyridine)$_2$](BF$_4$)$_2$ (3).**

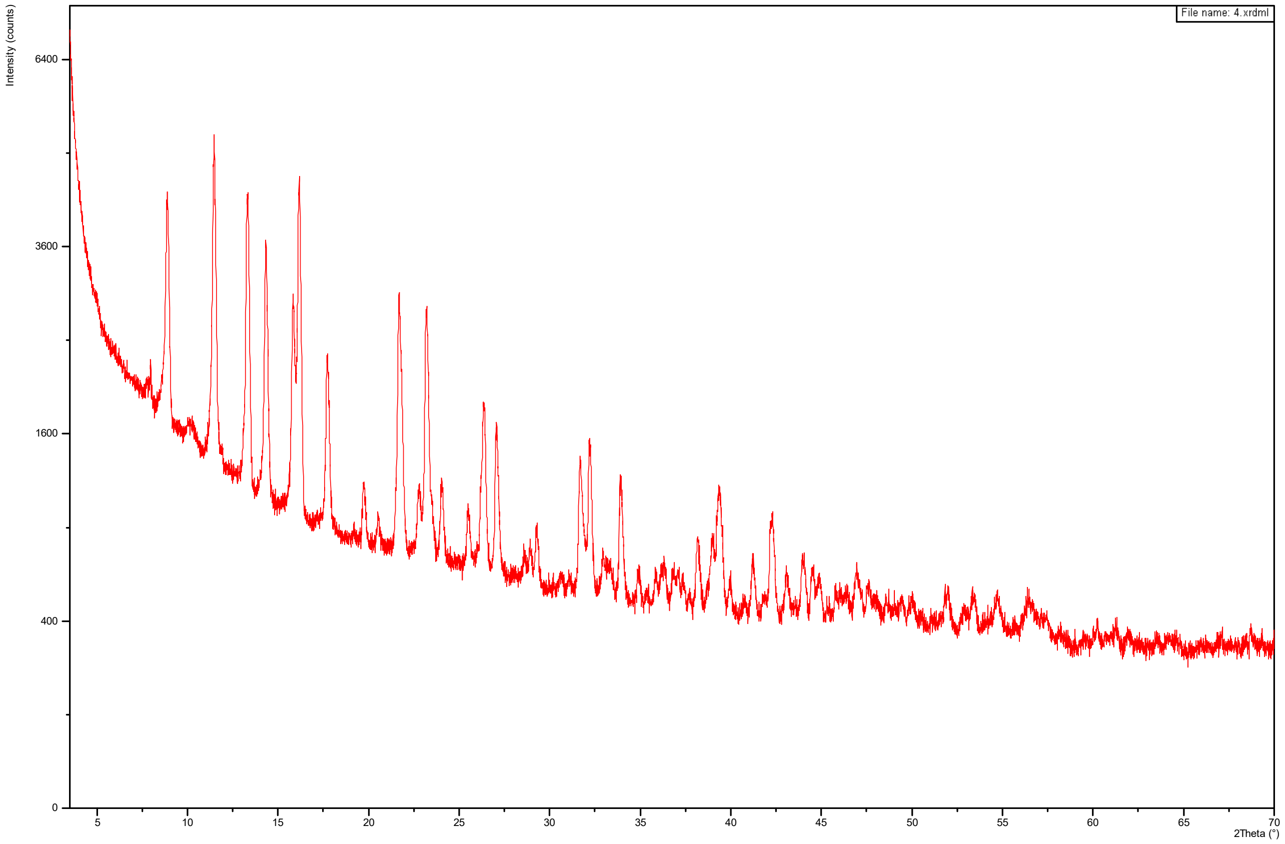


**Figure S 25. XRD of [Os (4'-(4-chlorophenyl)-2,2′:6′,2″-terpyridine)$_2$](BF$_4$)$_2$ (4).**

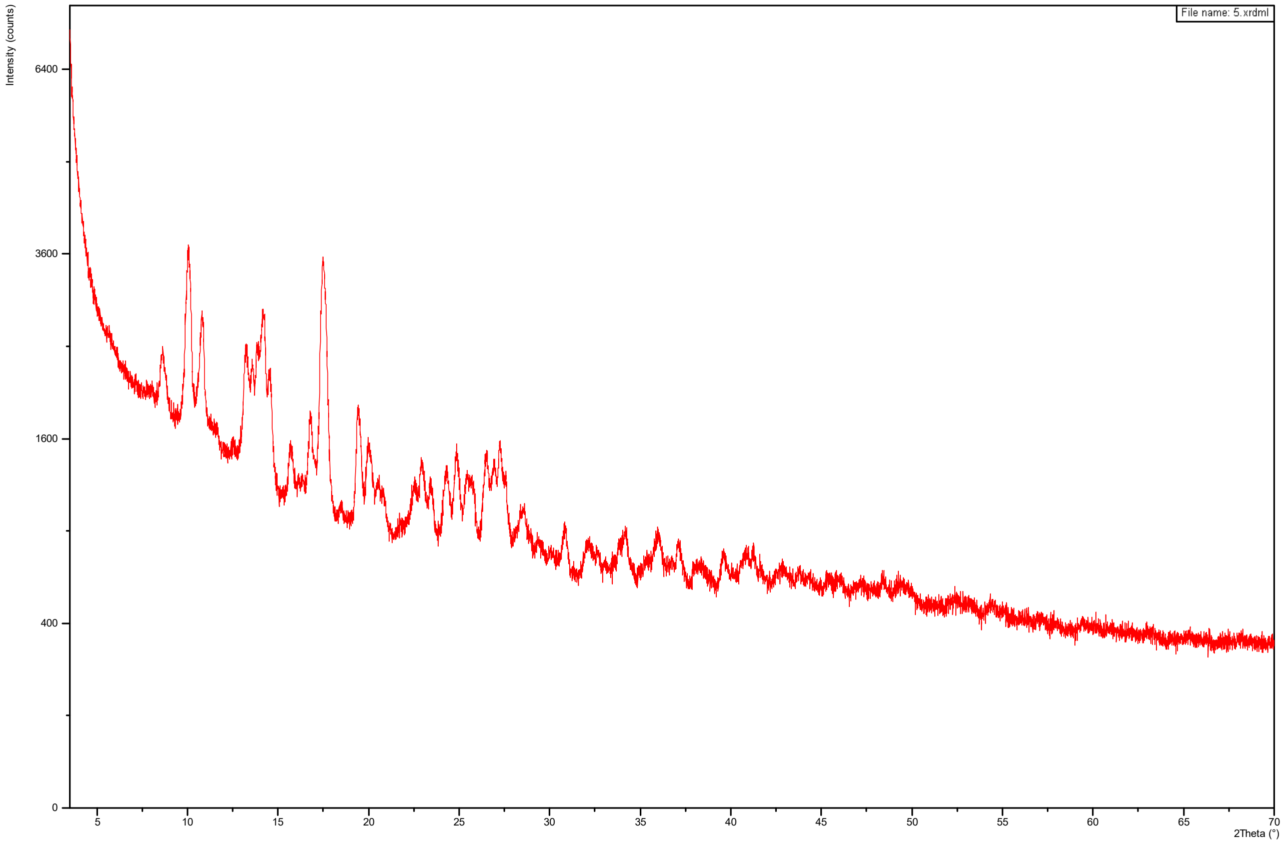


**Figure S 26. XRD of [Os (4'-chloro-2,2′:6′,2″-terpyridine)$_2$](BF$_4$)$_2$ (5).**

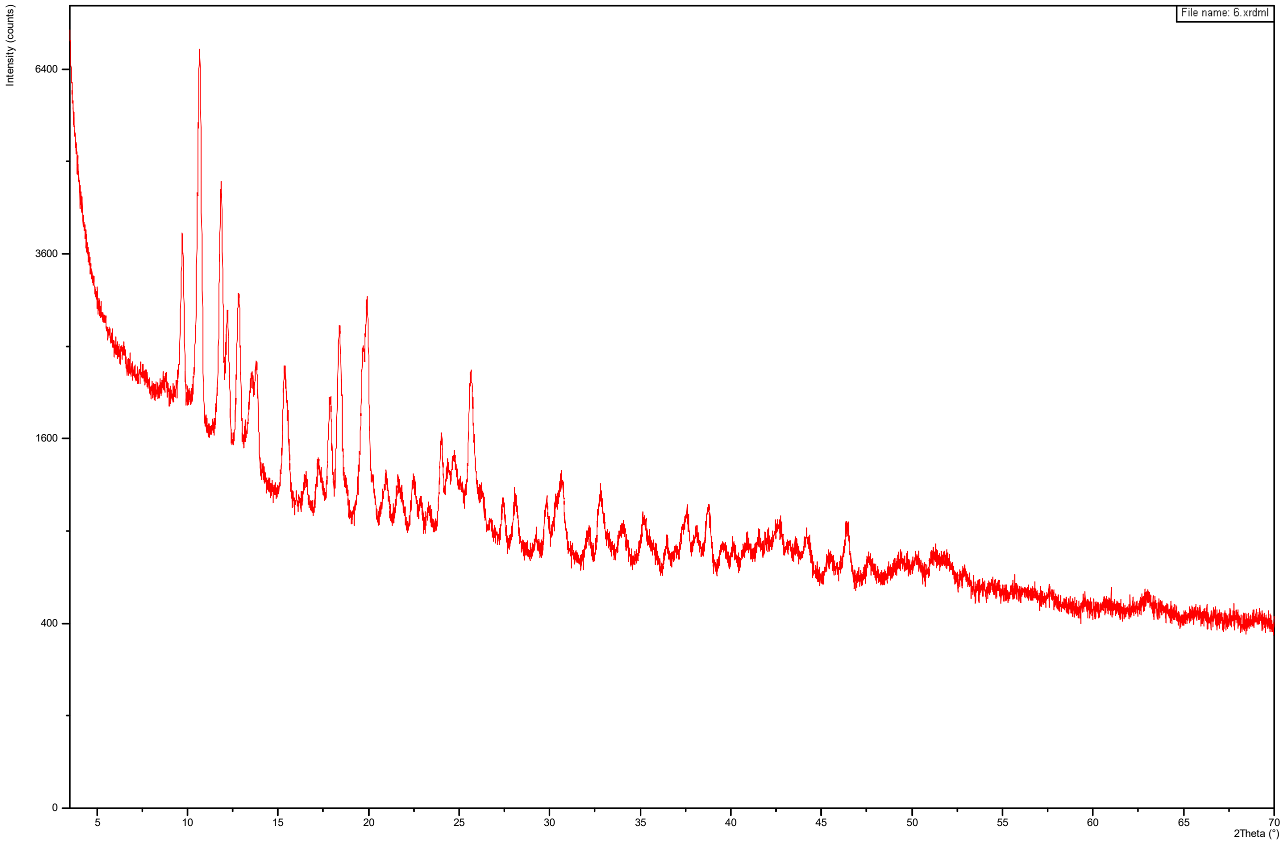


**Figure S 27. XRD of [Os (4'-methoxy)-2,2′:6′,2″-terpyridine)$_2$](BF$_4$)$_2$ (6).**

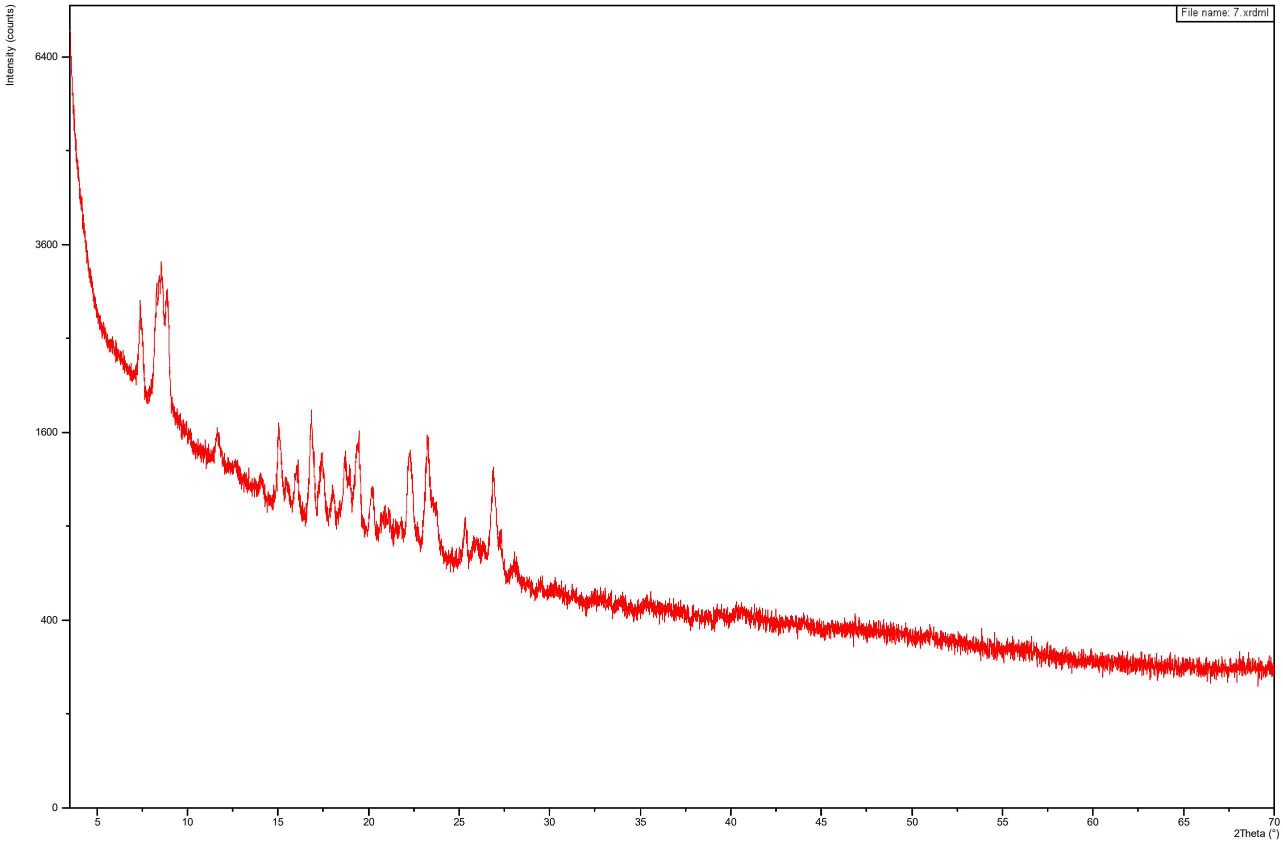


**Figure S 28. XRD of [Os 4'-(N-Pyrrolidinyl)-2,2′:6′,2″-terpyridine)$_2$](BF$_4$)$_2$ (7).**

## 6 Optimized coordinates

Density functional theory (DFT) calculations were performed using the B3LYP functional which is composed of the Becke 88 exchange functional [1] in combination with the LYP correlation functional [2], as implemented in the Gaussian 16 package [3]. The triple-ζ basis set 6-311G(d,p) was used for lighter atoms (C, H, F, O) and the def2-TZVPP basis set for both the core and valence electrons of Os. Optimizations were performed in $CH_3CN$ as solvent, using the implicit solvent Polarizable Continuum Model (PCM) [4] that uses the integral equation formalism variant (IEFPCM) [5].

### 6.1 [Os (2,2′:6′,2″-terpyridine)$_2$]$^{2+}$

| | | | |
|---|---|---|---|
| Os | 0.000000000 | 0.000000000 | 0.000000000 |
| N | 2.058234000 | 0.000000000 | 0.426799000 |
| N | 0.000000000 | 2.058234000 | -0.426799000 |
| N | 0.000000000 | 0.000000000 | 2.008594000 |
| N | 0.000000000 | 0.000000000 | -2.008594000 |
| N | -2.058234000 | 0.000000000 | 0.426799000 |
| N | 0.000000000 | -2.058234000 | -0.426799000 |
| C | 4.710669000 | 0.000000000 | 1.295888000 |
| C | 3.669992000 | 0.000000000 | 2.214926000 |
| C | 4.407073000 | 0.000000000 | -0.062079000 |
| C | 0.000000000 | 4.407073000 | 0.062079000 |
| C | 0.000000000 | 4.710669000 | -1.295888000 |
| C | 2.350347000 | 0.000000000 | 1.769797000 |
| C | 1.207023000 | 0.000000000 | 4.062426000 |
| C | 3.077807000 | 0.000000000 | -0.454232000 |
| C | 0.000000000 | 3.077807000 | 0.454232000 |
| C | 0.000000000 | 3.669992000 | -2.214926000 |
| C | 1.187818000 | 0.000000000 | 2.670056000 |
| C | 0.000000000 | 0.000000000 | 4.756563000 |
| C | 0.000000000 | 2.350347000 | -1.769797000 |
| C | -1.207023000 | 0.000000000 | 4.062426000 |
| C | 0.000000000 | 1.187818000 | -2.670056000 |
| C | 0.000000000 | 1.207023000 | -4.062426000 |
| C | -1.187818000 | 0.000000000 | 2.670056000 |
| C | 0.000000000 | 0.000000000 | -4.756563000 |
| C | -2.350347000 | 0.000000000 | 1.769797000 |
| C | 0.000000000 | -1.187818000 | -2.670056000 |
| C | 0.000000000 | -1.207023000 | -4.062426000 |
| C | -3.669992000 | 0.000000000 | 2.214926000 |
| C | 0.000000000 | -3.077807000 | 0.454232000 |
| C | 0.000000000 | -2.350347000 | -1.769797000 |
| C | -3.077807000 | 0.000000000 | -0.454232000 |
| C | -4.710669000 | 0.000000000 | 1.295888000 |
| C | 0.000000000 | -4.407073000 | 0.062079000 |
| C | 0.000000000 | -3.669992000 | -2.214926000 |

| | | | |
|---|---|---|---|
| C | -4.407073000 | 0.000000000 | -0.062079000 |
| C | 0.000000000 | -4.710669000 | -1.295888000 |
| H | 5.738526000 | 0.000000000 | 1.634997000 |
| H | 3.879909000 | 0.000000000 | 3.275344000 |
| H | 5.182841000 | 0.000000000 | -0.816007000 |
| H | 0.000000000 | 5.182841000 | 0.816007000 |
| H | 0.000000000 | 5.738526000 | -1.634997000 |
| H | 2.144247000 | 0.000000000 | 4.601106000 |
| H | 0.000000000 | 2.805292000 | 1.500365000 |
| H | 0.000000000 | 3.879909000 | -3.275344000 |
| H | 0.000000000 | 0.000000000 | 5.838597000 |
| H | 2.805292000 | 0.000000000 | -1.500365000 |
| H | 0.000000000 | 2.144247000 | -4.601106000 |
| H | -2.144247000 | 0.000000000 | 4.601106000 |
| H | 0.000000000 | 0.000000000 | -5.838597000 |
| H | 0.000000000 | -2.805292000 | 1.500365000 |
| H | -3.879909000 | 0.000000000 | 3.275344000 |
| H | 0.000000000 | -2.144247000 | -4.601106000 |
| H | -2.805292000 | 0.000000000 | -1.500365000 |
| H | 0.000000000 | -5.182841000 | 0.816007000 |
| H | 0.000000000 | -3.879909000 | -3.275344000 |
| H | -5.738526000 | 0.000000000 | 1.634997000 |
| H | -5.182841000 | 0.000000000 | -0.816007000 |
| H | 0.000000000 | -5.738526000 | -1.634997000 |

## 6.2 [Os (4'-(4-methylphenyl)-2,2′:6′,2″-terpyridine)$_2$]$^{2+}$

| | | | |
|---|---|---|---|
| Os | -0.000002000 | 0.003688000 | -0.001165000 |
| N | -0.430841000 | -1.620269000 | -1.266036000 |
| N | 0.429456000 | -1.260909000 | 1.623398000 |
| N | -2.006713000 | 0.003455000 | -0.001484000 |
| N | 2.006709000 | 0.002246000 | -0.000121000 |
| N | -0.431733000 | 1.628282000 | 1.262759000 |
| N | 0.433105000 | 1.267865000 | -1.625164000 |
| C | -1.300789000 | -3.710177000 | -2.898300000 |
| C | -2.219604000 | -2.891889000 | -2.255077000 |
| C | 0.057367000 | -3.469522000 | -2.714606000 |
| C | -0.060245000 | -2.707542000 | 3.473781000 |
| C | 1.297719000 | -2.892383000 | 3.714649000 |
| C | -1.774333000 | -1.851850000 | -1.442712000 |
| C | -4.062044000 | -0.950887000 | -0.733490000 |
| C | 0.449920000 | -2.422131000 | -1.896474000 |
| C | -0.451946000 | -1.889846000 | 2.425733000 |
| C | 2.217193000 | -2.250720000 | 2.895881000 |
| C | -2.673244000 | -0.934692000 | -0.722930000 |
| C | -4.786685000 | 0.000376000 | 0.001519000 |
| C | 1.772756000 | -1.438761000 | 1.855165000 |
| C | -4.062599000 | 0.953536000 | 0.734563000 |
| C | 2.672421000 | -0.720607000 | 0.937490000 |
| C | 4.061220000 | -0.734627000 | 0.952270000 |
| C | -2.673765000 | 0.940356000 | 0.721035000 |
| C | 4.786677000 | -0.001831000 | -0.000035000 |

| | | | |
|---|---|---|---|
| C | -1.775363000 | 1.859026000 | 1.439517000 |
| C | 2.674575000 | 0.723300000 | -0.937604000 |
| C | 4.063411000 | 0.733331000 | -0.952228000 |
| C | -2.221283000 | 2.899777000 | 2.250620000 |
| C | -0.446514000 | 1.898543000 | -2.428101000 |
| C | 1.776925000 | 1.443404000 | -1.855758000 |
| C | 0.448524000 | 2.431709000 | 1.891921000 |
| C | -1.302973000 | 3.719636000 | 2.892552000 |
| C | -0.052471000 | 2.715841000 | -3.475588000 |
| C | 2.223674000 | 2.254891000 | -2.895850000 |
| C | 0.055328000 | 3.479831000 | 2.708811000 |
| C | 1.306020000 | 2.898395000 | -3.715217000 |
| H | -1.640290000 | -4.519937000 | -3.531176000 |
| H | -3.279895000 | -3.059229000 | -2.383677000 |
| H | 0.810512000 | -4.079728000 | -3.194834000 |
| H | -0.813890000 | -3.186541000 | 4.084334000 |
| H | 1.636564000 | -3.524910000 | 4.524955000 |
| H | -4.587285000 | -1.685563000 | -1.326908000 |
| H | -1.498047000 | -1.724114000 | 2.208758000 |
| H | 3.277357000 | -2.380117000 | 3.063442000 |
| H | 1.496187000 | -2.205348000 | -1.731545000 |
| H | 4.585551000 | -1.329715000 | 1.686219000 |
| H | -4.587984000 | 1.686826000 | 1.329538000 |
| H | -1.493079000 | 1.734495000 | -2.212099000 |
| H | -3.281688000 | 3.066544000 | 2.379079000 |
| H | 4.589717000 | 1.326553000 | -1.686303000 |
| H | 1.494924000 | 2.215549000 | 1.727034000 |
| H | -0.804749000 | 3.196314000 | -4.086670000 |
| H | 3.284206000 | 2.382534000 | -3.062415000 |
| H | -1.642959000 | 4.529981000 | 3.524417000 |
| H | 0.808106000 | 4.091273000 | 3.188041000 |
| H | 1.646659000 | 3.530589000 | -4.525031000 |
| C | -6.266902000 | -0.002237000 | 0.003572000 |
| C | -6.991920000 | 1.197677000 | 0.061904000 |
| C | -6.987144000 | -1.204879000 | -0.047974000 |
| H | -6.469017000 | 2.146835000 | 0.081031000 |
| H | -6.460563000 | -2.152082000 | -0.063009000 |
| C | -8.381806000 | 1.191054000 | 0.064376000 |
| C | -8.377481000 | -1.203964000 | -0.039549000 |
| H | -8.915967000 | 2.134541000 | 0.102493000 |
| H | -8.907920000 | -2.149898000 | -0.064596000 |
| C | -9.102629000 | -0.008048000 | 0.012772000 |
| C | 6.266898000 | -0.004290000 | -0.000635000 |
| C | 6.988850000 | 0.046804000 | -1.202173000 |
| C | 6.990205000 | -0.062550000 | 1.200412000 |
| H | 6.463663000 | 0.061916000 | -2.150146000 |
| H | 6.465900000 | -0.081174000 | 2.148807000 |
| C | 8.379273000 | 0.037926000 | -1.199228000 |
| C | 8.380008000 | -0.065461000 | 1.195814000 |
| H | 8.911081000 | 0.062706000 | -2.144389000 |
| H | 8.912787000 | -0.103417000 | 2.140099000 |
| C | 9.102627000 | -0.014414000 | -0.002331000 |
| C | 10.610433000 | 0.007971000 | -0.000063000 |
| H | 11.015504000 | -0.383980000 | -0.935024000 |

| | | | |
|---|---|---|---|
| H | 10.982837000 | 1.031626000 | 0.115827000 |
| H | 11.015622000 | -0.580572000 | 0.825937000 |
| C | -10.610418000 | -0.008522000 | -0.010563000 |
| H | -11.017362000 | 0.827702000 | 0.561976000 |
| H | -10.982286000 | 0.086960000 | -1.036537000 |
| H | -11.014236000 | -0.936698000 | 0.398588000 |

## 6.3 [Os (4,4’,4"-tri-tert-Butyl-2,2′:6′,2′′-terpyridine)$_2$]$^{2+}$

| | | | |
|---|---|---|---|
| Os | -0.004223000 | -0.001959000 | 0.002156000 |
| N | -0.387192000 | -2.068889000 | 0.017713000 |
| N | 0.422188000 | 0.025140000 | 2.059694000 |
| N | -2.010615000 | -0.052509000 | -0.000298000 |
| N | 2.003120000 | 0.053772000 | 0.009867000 |
| N | -0.489693000 | 2.042417000 | -0.016929000 |
| N | 0.436296000 | -0.006344000 | -2.053278000 |
| C | -1.218039000 | -4.775020000 | 0.042743000 |
| C | -2.135852000 | -3.725138000 | 0.033577000 |
| C | 0.136512000 | -4.410035000 | 0.037680000 |
| C | -0.046419000 | 0.024281000 | 4.415163000 |
| C | 1.310852000 | 0.061829000 | 4.747562000 |
| C | -1.718523000 | -2.395642000 | 0.021458000 |
| C | -4.036173000 | -1.302408000 | 0.015751000 |
| C | 0.506058000 | -3.078928000 | 0.025427000 |
| C | -0.444258000 | 0.007164000 | 3.087361000 |
| C | 2.206788000 | 0.081804000 | 3.672774000 |
| C | -2.648355000 | -1.252812000 | 0.012143000 |
| C | -4.796948000 | -0.122692000 | 0.006512000 |
| C | 1.764528000 | 0.064340000 | 2.356279000 |
| C | -4.101250000 | 1.090282000 | -0.006574000 |
| C | 2.665977000 | 0.084583000 | 1.191442000 |
| C | 4.058326000 | 0.132383000 | 1.212918000 |
| C | -2.708262000 | 1.109417000 | -0.009929000 |
| C | 4.788622000 | 0.149158000 | 0.020680000 |
| C | -1.840641000 | 2.299984000 | -0.021319000 |
| C | 2.675290000 | 0.066257000 | -1.171044000 |
| C | 4.062785000 | 0.113636000 | -1.180513000 |
| C | -2.321028000 | 3.603151000 | -0.035402000 |
| C | -0.423609000 | -0.039001000 | -3.085987000 |
| C | 1.780166000 | 0.028486000 | -2.341687000 |
| C | 0.347024000 | 3.094414000 | -0.025824000 |
| C | -1.455880000 | 4.703142000 | -0.044751000 |
| C | -0.017334000 | -0.041111000 | -4.411376000 |
| C | 2.231028000 | 0.026836000 | -3.655110000 |
| C | -0.089036000 | 4.410153000 | -0.039387000 |
| C | 1.341925000 | -0.008582000 | -4.735399000 |
| H | -3.195556000 | -3.929293000 | 0.036167000 |
| H | 0.921107000 | -5.154900000 | 0.043633000 |
| H | -0.815400000 | 0.007075000 | 5.173548000 |
| H | -4.529561000 | -2.264009000 | 0.026434000 |
| H | -1.493636000 | -0.021604000 | 2.827105000 |
| H | 3.271235000 | 0.110591000 | 3.857273000 |

| | | | |
|---|---|---|---|
| H | 1.549202000 | -2.793622000 | 0.022029000 |
| H | 4.567952000 | 0.157882000 | 2.163705000 |
| H | -4.638872000 | 2.025781000 | -0.013105000 |
| H | -1.474595000 | -0.064034000 | -2.831909000 |
| H | -3.390840000 | 3.757343000 | -0.039305000 |
| H | 4.583623000 | 0.124888000 | -2.127417000 |
| H | 1.403757000 | 2.863919000 | -0.021771000 |
| H | -0.781405000 | -0.068953000 | -5.174401000 |
| H | 3.296662000 | 0.052910000 | -3.833436000 |
| H | 0.657994000 | 5.190445000 | -0.045565000 |
| C | 6.323071000 | 0.206289000 | -0.012829000 |
| C | -6.330873000 | -0.199701000 | 0.013887000 |
| C | 1.827363000 | 0.079157000 | 6.190969000 |
| C | 1.867712000 | -0.012560000 | -6.175499000 |
| C | -2.013751000 | 6.131064000 | -0.059699000 |
| C | -1.630593000 | -6.251035000 | 0.058483000 |
| C | 6.860857000 | -1.043759000 | -0.748729000 |
| H | 7.953190000 | -1.012271000 | -0.780112000 |
| H | 6.561366000 | -1.960151000 | -0.233336000 |
| H | 6.500463000 | -1.099912000 | -1.778297000 |
| C | 6.765453000 | 1.482287000 | -0.767723000 |
| H | 7.857054000 | 1.532248000 | -0.799671000 |
| H | 6.401777000 | 1.496396000 | -1.797599000 |
| H | 6.398221000 | 2.381037000 | -0.265247000 |
| C | 6.933241000 | 0.240371000 | 1.399638000 |
| H | 6.678588000 | -0.652991000 | 1.976167000 |
| H | 8.021867000 | 0.281133000 | 1.320561000 |
| H | 6.611141000 | 1.120699000 | 1.962351000 |
| C | -6.793651000 | -0.935660000 | 1.293638000 |
| H | -6.399495000 | -1.952958000 | 1.346148000 |
| H | -6.472217000 | -0.399962000 | 2.190757000 |
| H | -7.884884000 | -1.000074000 | 1.308264000 |
| C | -6.805911000 | -0.981372000 | -1.233742000 |
| H | -6.491190000 | -0.479933000 | -2.152746000 |
| H | -6.415018000 | -2.001090000 | -1.252264000 |
| H | -7.897352000 | -1.043643000 | -1.236627000 |
| C | -6.981850000 | 1.194668000 | -0.008482000 |
| H | -6.707760000 | 1.787508000 | 0.868308000 |
| H | -6.711765000 | 1.756904000 | -0.906468000 |
| H | -8.068530000 | 1.084582000 | -0.003795000 |
| C | -2.884445000 | 6.346168000 | 1.201071000 |
| H | -3.727871000 | 5.653125000 | 1.239157000 |
| H | -2.294061000 | 6.214485000 | 2.111657000 |
| H | -3.287759000 | 7.362235000 | 1.200818000 |
| C | -1.053683000 | -6.919577000 | 1.329008000 |
| H | -1.335164000 | -7.975688000 | 1.348747000 |
| H | 0.036576000 | -6.863977000 | 1.361144000 |
| H | -1.445557000 | -6.445790000 | 2.232909000 |
| C | -3.159041000 | -6.428784000 | 0.062216000 |
| H | -3.622448000 | -5.997555000 | -0.829225000 |
| H | -3.397461000 | -7.494664000 | 0.073717000 |
| H | -3.620277000 | -5.978892000 | 0.945539000 |
| C | -1.056614000 | -6.946616000 | -1.198716000 |
| H | 0.033562000 | -6.891806000 | -1.234558000 |

| | | | |
|---|---|---|---|
| H | -1.338075000 | -8.002926000 | -1.195165000 |
| H | -1.450594000 | -6.492336000 | -2.111659000 |
| C | -0.894012000 | 7.186514000 | -0.068623000 |
| H | -0.261855000 | 7.116816000 | 0.820518000 |
| H | -0.258091000 | 7.097776000 | -0.953349000 |
| H | -1.338556000 | 8.184200000 | -0.080233000 |
| C | -2.881077000 | 6.321045000 | -1.326779000 |
| H | -2.288128000 | 6.172047000 | -2.233007000 |
| H | -3.724068000 | 5.626924000 | -1.353694000 |
| H | -3.284947000 | 7.336664000 | -1.347387000 |
| C | 2.665826000 | 1.360284000 | 6.413367000 |
| H | 3.037267000 | 1.382795000 | 7.441246000 |
| H | 3.529782000 | 1.405437000 | 5.746515000 |
| H | 2.061996000 | 2.256752000 | 6.249901000 |
| C | 2.714642000 | -1.166976000 | 6.423572000 |
| H | 2.145304000 | -2.087384000 | 6.269319000 |
| H | 3.578198000 | -1.184766000 | 5.754830000 |
| H | 3.088591000 | -1.166034000 | 7.450819000 |
| C | 0.677858000 | 0.061572000 | 7.213914000 |
| H | 0.064793000 | -0.838848000 | 7.122283000 |
| H | 1.093460000 | 0.076391000 | 8.223955000 |
| H | 0.028119000 | 0.934707000 | 7.111016000 |
| C | 0.724701000 | -0.042869000 | -7.205388000 |
| H | 1.146684000 | -0.043706000 | -8.212888000 |
| H | 0.109190000 | -0.940591000 | -7.104400000 |
| H | 0.076132000 | 0.832962000 | -7.119380000 |
| C | 2.754212000 | -1.263454000 | -6.384308000 |
| H | 3.612647000 | -1.273718000 | -5.708830000 |
| H | 2.181853000 | -2.180539000 | -6.221707000 |
| H | 3.135860000 | -1.277386000 | -7.408612000 |
| C | 2.709765000 | 1.263863000 | -6.410871000 |
| H | 3.569992000 | 1.316797000 | -5.739800000 |
| H | 3.087075000 | 1.271429000 | -7.436835000 |
| H | 2.106641000 | 2.163568000 | -6.263333000 |

## 6.4 [Os (4'-(4-chlorophenyl)-2,2′:6′,2″-terpyridine)$_2$]$^{2+}$

| | | | |
|---|---|---|---|
| Os | 0.000000000 | 0.000000000 | -0.000001000 |
| N | -2.047130000 | 0.214368000 | 0.430135000 |
| N | -0.214372000 | -2.047129000 | -0.430136000 |
| N | 0.000000000 | 0.000000000 | 2.006021000 |
| N | 0.000000000 | 0.000000000 | -2.006023000 |
| N | 2.047130000 | -0.214368000 | 0.430135000 |
| N | 0.214372000 | 2.047129000 | -0.430136000 |
| C | -4.684543000 | 0.489676000 | 1.299602000 |
| C | -3.649838000 | 0.378938000 | 2.218721000 |
| C | -4.382884000 | 0.461142000 | -0.058475000 |
| C | -0.461150000 | -4.382882000 | 0.058475000 |
| C | -0.489680000 | -4.684542000 | -1.299602000 |
| C | -2.337311000 | 0.242260000 | 1.773474000 |
| C | -1.196908000 | 0.121269000 | 4.061021000 |
| C | -3.060991000 | 0.322914000 | -0.451011000 |

| | | | |
|---|---|---|---|
| C | -0.322922000 | -3.060989000 | 0.451010000 |
| C | -0.378937000 | -3.649838000 | -2.218721000 |
| C | -1.178121000 | 0.118403000 | 2.672062000 |
| C | 0.000000000 | 0.000000000 | 4.782279000 |
| C | -0.242257000 | -2.337311000 | -1.773475000 |
| C | 1.196908000 | -0.121269000 | 4.061021000 |
| C | -0.118397000 | -1.178122000 | -2.672064000 |
| C | -0.121260000 | -1.196910000 | -4.061023000 |
| C | 1.178121000 | -0.118403000 | 2.672062000 |
| C | 0.000000000 | 0.000000000 | -4.782279000 |
| C | 2.337311000 | -0.242260000 | 1.773474000 |
| C | 0.118397000 | 1.178122000 | -2.672064000 |
| C | 0.121260000 | 1.196910000 | -4.061023000 |
| C | 3.649838000 | -0.378938000 | 2.218721000 |
| C | 0.322922000 | 3.060989000 | 0.451010000 |
| C | 0.242257000 | 2.337311000 | -1.773475000 |
| C | 3.060991000 | -0.322914000 | -0.451011000 |
| C | 4.684543000 | -0.489676000 | 1.299602000 |
| C | 0.461150000 | 4.382882000 | 0.058475000 |
| C | 0.378937000 | 3.649838000 | -2.218721000 |
| C | 4.382884000 | -0.461142000 | -0.058475000 |
| C | 0.489680000 | 4.684542000 | -1.299602000 |
| H | -5.706767000 | 0.596209000 | 1.638929000 |
| H | -3.860658000 | 0.398697000 | 3.278876000 |
| H | -5.154827000 | 0.543868000 | -0.811732000 |
| H | -0.543881000 | -5.154824000 | 0.811732000 |
| H | -0.596216000 | -5.706766000 | -1.638928000 |
| H | -2.133275000 | 0.239886000 | 4.587335000 |
| H | -0.296500000 | -2.790222000 | 1.497407000 |
| H | -0.398695000 | -3.860658000 | -3.278876000 |
| H | -2.790226000 | 0.296490000 | -1.497408000 |
| H | -0.239883000 | -2.133277000 | -4.587338000 |
| H | 2.133275000 | -0.239886000 | 4.587335000 |
| H | 0.296500000 | 2.790222000 | 1.497407000 |
| H | 3.860658000 | -0.398697000 | 3.278876000 |
| H | 0.239883000 | 2.133277000 | -4.587338000 |
| H | 2.790226000 | -0.296490000 | -1.497408000 |
| H | 0.543881000 | 5.154824000 | 0.811732000 |
| H | 0.398695000 | 3.860658000 | -3.278876000 |
| H | 5.706767000 | -0.596209000 | 1.638929000 |
| H | 5.154827000 | -0.543868000 | -0.811732000 |
| H | 0.596216000 | 5.706766000 | -1.638928000 |
| C | 0.000000000 | 0.000000000 | 6.263729000 |
| C | 1.030402000 | 0.624286000 | 6.981743000 |
| C | -1.030402000 | -0.624286000 | 6.981743000 |
| H | 1.827350000 | 1.136737000 | 6.456832000 |
| H | -1.827350000 | -1.136737000 | 6.456832000 |
| C | 1.035517000 | 0.630083000 | 8.372116000 |
| C | -1.035517000 | -0.630083000 | 8.372116000 |
| H | 1.829464000 | 1.124593000 | 8.915998000 |
| H | -1.829464000 | -1.124593000 | 8.915998000 |
| C | 0.000000000 | 0.000000000 | 9.053895000 |
| C | 0.000000000 | 0.000000000 | -6.263728000 |
| C | -0.624325000 | 1.030378000 | -6.981740000 |

| | | | |
|---|---|---|---|
| C | 0.624325000 | -1.030378000 | -6.981740000 |
| H | -1.136808000 | 1.827304000 | -6.456828000 |
| H | 1.136808000 | -1.827304000 | -6.456828000 |
| C | -0.630122000 | 1.035493000 | -8.372114000 |
| C | 0.630122000 | -1.035493000 | -8.372114000 |
| H | -1.124666000 | 1.829418000 | -8.915995000 |
| H | 1.124666000 | -1.829418000 | -8.915995000 |
| C | 0.000000000 | 0.000000000 | -9.053892000 |
| Cl | 0.000000000 | 0.000000000 | 10.815313000 |
| Cl | 0.000000000 | 0.000000000 | -10.815310000 |

### 6.5 [Os (4’-chloro-2,2′:6′,2″-terpyridine)$_2$]$^{2+}$

| | | | |
|---|---|---|---|
| Os | 0.000000000 | 0.000000000 | 0.000000000 |
| N | 2.059438000 | 0.000000000 | 0.429112000 |
| N | 0.000000000 | 2.059438000 | -0.429112000 |
| N | 0.000000000 | 0.000000000 | 2.008442000 |
| N | 0.000000000 | 0.000000000 | -2.008442000 |
| N | -2.059438000 | 0.000000000 | 0.429112000 |
| N | 0.000000000 | -2.059438000 | -0.429112000 |
| C | 4.711487000 | 0.000000000 | 1.302941000 |
| C | 3.669235000 | 0.000000000 | 2.220673000 |
| C | 4.409385000 | 0.000000000 | -0.054720000 |
| C | 0.000000000 | 4.409385000 | 0.054720000 |
| C | 0.000000000 | 4.711487000 | -1.302941000 |
| C | 2.351462000 | 0.000000000 | 1.772372000 |
| C | 1.212865000 | 0.000000000 | 4.060982000 |
| C | 3.080366000 | 0.000000000 | -0.449531000 |
| C | 0.000000000 | 3.080366000 | 0.449531000 |
| C | 0.000000000 | 3.669235000 | -2.220673000 |
| C | 1.186614000 | 0.000000000 | 2.670211000 |
| C | 0.000000000 | 0.000000000 | 4.743115000 |
| C | 0.000000000 | 2.351462000 | -1.772372000 |
| C | -1.212865000 | 0.000000000 | 4.060982000 |
| C | 0.000000000 | 1.186614000 | -2.670211000 |
| C | 0.000000000 | 1.212865000 | -4.060982000 |
| C | -1.186614000 | 0.000000000 | 2.670211000 |
| C | 0.000000000 | 0.000000000 | -4.743115000 |
| C | -2.351462000 | 0.000000000 | 1.772372000 |
| C | 0.000000000 | -1.186614000 | -2.670211000 |
| C | 0.000000000 | -1.212865000 | -4.060982000 |
| C | -3.669235000 | 0.000000000 | 2.220673000 |
| C | 0.000000000 | -3.080366000 | 0.449531000 |
| C | 0.000000000 | -2.351462000 | -1.772372000 |
| C | -3.080366000 | 0.000000000 | -0.449531000 |
| C | -4.711487000 | 0.000000000 | 1.302941000 |
| C | 0.000000000 | -4.409385000 | 0.054720000 |
| C | 0.000000000 | -3.669235000 | -2.220673000 |
| C | -4.409385000 | 0.000000000 | -0.054720000 |
| C | 0.000000000 | -4.711487000 | -1.302941000 |
| H | 5.738763000 | 0.000000000 | 1.643564000 |
| H | 3.878944000 | 0.000000000 | 3.281060000 |

| | | | |
|---|---|---|---|
| H | 5.186107000 | 0.000000000 | -0.807587000 |
| H | 0.000000000 | 5.186107000 | 0.807587000 |
| H | 0.000000000 | 5.738763000 | -1.643564000 |
| H | 2.145696000 | 0.000000000 | 4.605099000 |
| H | 0.000000000 | 2.811024000 | 1.496298000 |
| H | 0.000000000 | 3.878944000 | -3.281060000 |
| H | 2.811024000 | 0.000000000 | -1.496298000 |
| H | 0.000000000 | 2.145696000 | -4.605099000 |
| H | -2.145696000 | 0.000000000 | 4.605099000 |
| H | 0.000000000 | -2.811024000 | 1.496298000 |
| H | -3.878944000 | 0.000000000 | 3.281060000 |
| H | 0.000000000 | -2.145696000 | -4.605099000 |
| H | -2.811024000 | 0.000000000 | -1.496298000 |
| H | 0.000000000 | -5.186107000 | 0.807587000 |
| H | 0.000000000 | -3.878944000 | -3.281060000 |
| H | -5.738763000 | 0.000000000 | 1.643564000 |
| H | -5.186107000 | 0.000000000 | -0.807587000 |
| H | 0.000000000 | -5.738763000 | -1.643564000 |
| Cl | 0.000000000 | 0.000000000 | -6.488830000 |
| Cl | 0.000000000 | 0.000000000 | 6.488830000 |

### 6.6 [Os (4’-methoxy)-2,2′:6′,2′′-terpyridine)$_2$]$^{2+}$

| | | | |
|---|---|---|---|
| Os | 0.000000000 | 0.000000000 | 0.036790000 |
| N | 1.402467000 | 0.590992000 | -1.413443000 |
| N | -1.402467000 | -0.590992000 | -1.413443000 |
| N | -0.216793000 | 2.001182000 | 0.036218000 |
| N | 0.216793000 | -2.001182000 | 0.036218000 |
| N | -1.493111000 | 0.269761000 | 1.491978000 |
| N | 1.493111000 | -0.269761000 | 1.491978000 |
| C | 3.186062000 | 1.666180000 | -3.274653000 |
| C | 2.350332000 | 2.496885000 | -2.540336000 |
| C | 3.117346000 | 0.292011000 | -3.066606000 |
| C | -3.117346000 | -0.292011000 | -3.066606000 |
| C | -3.186062000 | -1.666180000 | -3.274653000 |
| C | 1.464009000 | 1.950789000 | -1.615082000 |
| C | 0.409734000 | 4.140686000 | -0.818164000 |
| C | 2.219503000 | -0.202768000 | -2.134115000 |
| C | -2.219503000 | 0.202768000 | -2.134115000 |
| C | -2.350332000 | -2.496885000 | -2.540336000 |
| C | 0.541910000 | 2.754379000 | -0.794474000 |
| C | -0.522213000 | 4.742974000 | 0.035413000 |
| C | -1.464009000 | -1.950789000 | -1.615082000 |
| C | -1.296572000 | 3.942315000 | 0.891080000 |
| C | -0.541910000 | -2.754379000 | -0.794474000 |
| C | -0.409734000 | -4.140686000 | -0.818164000 |
| C | -1.126033000 | 2.569755000 | 0.873735000 |
| C | 0.522213000 | -4.742974000 | 0.035413000 |
| C | -1.846474000 | 1.582668000 | 1.697090000 |
| C | 1.126033000 | -2.569755000 | 0.873735000 |
| C | 1.296572000 | -3.942315000 | 0.891080000 |
| C | -2.823441000 | 1.924307000 | 2.628362000 |

| | | | |
|---|---|---|---|
| C | 2.114643000 | 0.682954000 | 2.214743000 |
| C | 1.846474000 | -1.582668000 | 1.697090000 |
| C | -2.114643000 | -0.682954000 | 2.214743000 |
| C | -3.455242000 | 0.931671000 | 3.365785000 |
| C | 3.092665000 | 0.394778000 | 3.153604000 |
| C | 2.823441000 | -1.924307000 | 2.628362000 |
| C | -3.092665000 | -0.394778000 | 3.153604000 |
| C | 3.455242000 | -0.931671000 | 3.365785000 |
| H | 3.876930000 | 2.085134000 | -3.994843000 |
| H | 2.387191000 | 3.567608000 | -2.684770000 |
| H | 3.747518000 | -0.396035000 | -3.614132000 |
| H | -3.747518000 | 0.396035000 | -3.614132000 |
| H | -3.876930000 | -2.085134000 | -3.994843000 |
| H | 1.016675000 | 4.732843000 | -1.484916000 |
| H | -2.139845000 | 1.264473000 | -1.945883000 |
| H | -2.387191000 | -3.567608000 | -2.684770000 |
| H | 2.139845000 | -1.264473000 | -1.945883000 |
| H | -1.016675000 | -4.732843000 | -1.484916000 |
| H | -2.010775000 | 4.422451000 | 1.544721000 |
| H | 1.808491000 | 1.702062000 | 2.023940000 |
| H | -3.087151000 | 2.962601000 | 2.774516000 |
| H | 2.010775000 | -4.422451000 | 1.544721000 |
| H | -1.808491000 | -1.702062000 | 2.023940000 |
| H | 3.555852000 | 1.203189000 | 3.703354000 |
| H | 3.087151000 | -2.962601000 | 2.774516000 |
| H | -4.215507000 | 1.190542000 | 4.091379000 |
| H | -3.555852000 | -1.203189000 | 3.703354000 |
| H | 4.215507000 | -1.190542000 | 4.091379000 |
| O | 0.748870000 | -6.064984000 | 0.110903000 |
| O | -0.748870000 | 6.064984000 | 0.110903000 |
| C | 0.000000000 | -6.953211000 | -0.733409000 |
| H | 0.187232000 | -6.737795000 | -1.788132000 |
| H | 0.360193000 | -7.950261000 | -0.494064000 |
| H | -1.069009000 | -6.886726000 | -0.517530000 |
| C | 0.000000000 | 6.953211000 | -0.733409000 |
| H | -0.360193000 | 7.950261000 | -0.494064000 |
| H | 1.069009000 | 6.886726000 | -0.517530000 |
| H | -0.187232000 | 6.737795000 | -1.788132000 |

## 6.7 [Os 4'-(N-Pyrrolidinyl)-2,2′:6′,2″-terpyridine)$_2$]$^{2+}$

| | | | |
|---|---|---|---|
| Os | 0.000000000 | 0.000000000 | 0.000000000 |
| N | 1.454163000 | 1.452826000 | 0.436843000 |
| C | 1.661523000 | 1.656973000 | 1.782512000 |
| C | 2.597835000 | 2.588092000 | 2.225375000 |
| C | 3.335060000 | 3.325090000 | 1.308018000 |
| C | 3.119230000 | 3.113699000 | -0.050353000 |
| C | 2.177497000 | 2.175778000 | -0.441963000 |
| N | 0.000000000 | 0.000000000 | 2.016854000 |
| C | 0.836061000 | 0.832540000 | 2.687248000 |
| C | 0.857958000 | 0.856957000 | 4.070777000 |
| C | 0.000000000 | 0.000000000 | 4.809324000 |

| | | | |
|---|---|---|---|
| C | -0.857958000 | -0.856957000 | 4.070777000 |
| C | -0.836061000 | -0.832540000 | 2.687248000 |
| N | -1.454163000 | -1.452826000 | 0.436843000 |
| C | -1.661523000 | -1.656973000 | 1.782512000 |
| C | -2.597835000 | -2.588092000 | 2.225375000 |
| C | -3.335060000 | -3.325090000 | 1.308018000 |
| C | -3.119230000 | -3.113699000 | -0.050353000 |
| C | -2.177497000 | -2.175778000 | -0.441963000 |
| N | 1.454163000 | -1.452826000 | -0.436843000 |
| C | 1.661523000 | -1.656973000 | -1.782512000 |
| C | 2.597835000 | -2.588092000 | -2.225375000 |
| C | 3.335060000 | -3.325090000 | -1.308018000 |
| C | 3.119229000 | -3.113699000 | 0.050353000 |
| C | 2.177496000 | -2.175778000 | 0.441963000 |
| N | 0.000000000 | 0.000000000 | -2.016854000 |
| C | 0.836060000 | -0.832540000 | -2.687248000 |
| C | 0.857957000 | -0.856957000 | -4.070777000 |
| C | 0.000000000 | 0.000000000 | -4.809324000 |
| C | -0.857957000 | 0.856957000 | -4.070777000 |
| C | -0.836060000 | 0.832540000 | -2.687248000 |
| N | -1.454163000 | 1.452826000 | -0.436843000 |
| C | -1.661523000 | 1.656973000 | -1.782512000 |
| C | -2.597835000 | 2.588092000 | -2.225375000 |
| C | -3.335060000 | 3.325090000 | -1.308018000 |
| C | -3.119229000 | 3.113699000 | 0.050353000 |
| C | -2.177496000 | 2.175778000 | 0.441963000 |
| H | 2.749914000 | 2.735485000 | 3.285442000 |
| H | 4.063480000 | 4.049654000 | 1.648822000 |
| H | 3.668488000 | 3.662930000 | -0.803499000 |
| H | 1.982624000 | 1.983573000 | -1.488018000 |
| H | 1.519538000 | 1.533835000 | 4.588468000 |
| H | -1.519538000 | -1.533835000 | 4.588468000 |
| H | -2.749914000 | -2.735485000 | 3.285442000 |
| H | -4.063480000 | -4.049654000 | 1.648822000 |
| H | -3.668488000 | -3.662930000 | -0.803499000 |
| H | -1.982624000 | -1.983573000 | -1.488018000 |
| H | 2.749914000 | -2.735485000 | -3.285442000 |
| H | 4.063480000 | -4.049654000 | -1.648822000 |
| H | 3.668488000 | -3.662930000 | 0.803499000 |
| H | 1.982624000 | -1.983574000 | 1.488018000 |
| H | 1.519537000 | -1.533836000 | -4.588468000 |
| H | -1.519537000 | 1.533836000 | -4.588468000 |
| H | -2.749914000 | 2.735485000 | -3.285442000 |
| H | -4.063480000 | 4.049654000 | -1.648822000 |
| H | -3.668488000 | 3.662930000 | 0.803499000 |
| H | -1.982624000 | 1.983574000 | 1.488018000 |
| N | 0.000000000 | 0.000000000 | -6.162177000 |
| C | -0.924948000 | 0.797701000 | -6.983167000 |
| H | -0.661032000 | 1.861504000 | -6.935798000 |
| H | -1.953139000 | 0.686884000 | -6.629610000 |
| H | -0.946301000 | 0.974078000 | -9.165351000 |
| C | -0.731395000 | 0.232924000 | -8.394995000 |
| H | -1.398907000 | -0.619475000 | -8.548170000 |
| C | 0.731395000 | -0.232924000 | -8.394995000 |

| | | | |
|---|---|---|---|
| H | 1.398907000 | 0.619475000 | -8.548170000 |
| H | 0.946301000 | -0.974078000 | -9.165351000 |
| C | 0.924948000 | -0.797701000 | -6.983167000 |
| H | 1.953139000 | -0.686884000 | -6.629610000 |
| H | 0.661032000 | -1.861504000 | -6.935798000 |
| N | 0.000000000 | 0.000000000 | 6.162177000 |
| C | 0.924948000 | 0.797701000 | 6.983166000 |
| H | 0.661032000 | 1.861504000 | 6.935798000 |
| H | 1.953140000 | 0.686884000 | 6.629610000 |
| H | 0.946301000 | 0.974078000 | 9.165351000 |
| C | 0.731395000 | 0.232924000 | 8.394995000 |
| H | 1.398907000 | -0.619475000 | 8.548170000 |
| C | -0.731395000 | -0.232924000 | 8.394995000 |
| H | -1.398907000 | 0.619475000 | 8.548170000 |
| H | -0.946301000 | -0.974078000 | 9.165351000 |
| C | -0.924948000 | -0.797701000 | 6.983166000 |
| H | -1.953140000 | -0.686884000 | 6.629610000 |
| H | -0.661032000 | -1.861504000 | 6.935798000 |

# 7 References


[1] A.D. Becke, Density-functional exchange-energy approximation with correct asymptotic behavior, Phys. Rev. A. 38 (1988) 3098–3100. https://doi.org/10.1103/PhysRevA.38.3098.

[2] C. Lee, W. Yang, R.G. Parr, Development of the Colle-Salvetti correlation-energy formula into a functional of the electron density, Phys. Rev. B. 37 (1988) 785–789. https://doi.org/10.1103/PhysRevB.37.785.

[3] M.J. Frisch, G.W. Trucks, H.B. Schlegel, G.E. Scuseria, M.A. Robb, J.R. Cheeseman, G. Scalmani, V. Barone, G.A. Petersson, H. Nakatsuji, X. Li, M. Caricato, A. V. Marenich, J. Bloino, B.G. Janesko, R. Gomperts, B. Mennucci, H.P. Hratchian, J. V. Ortiz, A.F. Izmaylov, J.L. Sonnenberg, D. Williams-Young, F. Ding, F. Lipparini, F. Egidi, J. Goings, B. Peng, A. Petrone, T. Henderson, D. Ranasinghe, V.G. Zakrzewski, J. Gao, N. Rega, G. Zheng, W. Liang, M. Hada, M. Ehara, K. Toyota, R. Fukuda, J. Hasegawa, M. Ishida, T. Nakajima, Y. Honda, O. Kitao, H. Nakai, T. Vreven, K. Throssell, J. Montgomery, J. A., J.E. Peralta, F. Ogliaro, M.J. Bearpark, J.J. Heyd, E.N. Brothers, K.N. Kudin, V.N. Staroverov, T.A. Keith, R. Kobayashi, J. Normand, K. Raghavachari, A.P. Rendell, J.C. Burant, S.S. Iyengar, J. Tomasi, M. Cossi, J.M. Millam, M. Klene, C. Adamo, R. Cammi, J.W. Ochterski, R.L. Martin, K. Morokuma, O. Farkas, J.B. Foresman, D.J. Fox, Gaussian 16, Revision B.01, (2016).

[4] A. V. Marenich, C.J. Cramer, D.G. Truhlar, Universal Solvation Model Based on Solute Electron Density and on a Continuum Model of the Solvent Defined by the Bulk Dielectric Constant and Atomic Surface Tensions, J. Phys. Chem. B. 113 (2009) 6378–6396. https://doi.org/10.1021/jp810292n.

[5] R.E. Skyner, J.L. Mcdonagh, C.R. Groom, T. Van Mourik, A review of methods for the calculation of solution free energies and the modelling of systems in solution, Phys. Chem. Chem. Phys. 17 (2015) 6174–6191. https://doi.org/10.1039/C5CP00288E.
